# Collective consciousness and its pathologies: Understanding the failure of AIDS control and treatment in the United States

**DOI:** 10.1186/1742-4682-4-10

**Published:** 2007-02-26

**Authors:** Rodrick M Wallace, Mindy T Fullilove, Robert E Fullilove, Deborah N Wallace

**Affiliations:** 1 The New York State Psychiatric Institute, 1051 Riverside Drive, New York, NY, 10032, USA; 2 Joseph L. Mailman School of Public Health, Columbia University, 722 W. 168 St., New York, NY, 10032, USA; 3 Consumers Union, 101 Truman Ave., Yonkers, NY, 10703, USA

## Abstract

We address themes of distributed cognition by extending recent formal developments in the theory of individual consciousness. While single minds appear biologically limited to one dynamic structure of linked cognitive submodules instantiating consciousness, organizations, by contrast, can support several, sometimes many, such constructs simultaneously, although these usually operate relatively slowly. System behavior remains, however, constrained not only by culture, but by a developmental path dependence generated by organizational history, in the context of market selection pressures. Such highly parallel multitasking – essentially an institutional collective consciousness – while capable of reducing inattentional blindness and the consequences of failures within individual workspaces, does not eliminate them, and introduces new characteristic malfunctions involving the distortion of information sent between workspaces and the possibility of pathological resilience – dysfunctional institutional lock-in. Consequently, organizations remain subject to canonical and idiosyncratic failures analogous to, but more complicated than, those afflicting individuals. Remediation is made difficult by the manner in which pathological externalities can write images of themselves onto both institutional function and corrective intervention. The perspective is applied to the failure of AIDS control and treatment in the United States.

## Background

Small, disciplined groups of humans are the most fearsome predators on Earth. In large-scale organization, we have recast even the topography and ecological dynamics of the planet. Our institutions, at all scales, are cognitive, taking the perspectives of Baars [1] and of Atlan and Cohen [2], in that they perceive patterns of threat or opportunity, compare those patterns with some internal, learned or inherited, picture of the world, and then choose one or a small number of responses from a much larger repertory of possibilities.

Both individuals and institutions operate within the constraints and affordances of culture, which, to take the perspective of the evolutionary anthropologist Robert Boyd, at the individual level, "...is as much a part of human biology as the enamel on our teeth..." (e.g. [3]).

One starting point for understanding the necessity of including culture in the study of cognition or consciousness at any scale lies in the observations of Nisbett et al. [4], and others, following the tradition of Markus and Kitayama [5], regarding fundamental differences in perception between test subjects of Southeast Asian and Western cultural heritage across an broad realm of experiments. East Asian perspectives are characterized as holistic and Western as analytic. Nisbett et al. [4] find:

(1) Social organization directs attention to some aspects of the perceptual field at the expense of others.

(2) What is attended to influences metaphysics.

(3) Metaphysics guides tacit epistemology, that is, beliefs about the nature of the world and causality.

(4) Epistemology dictates the development and application of some cognitive processes at the expense of others.

(5) Social organization can directly affect the plausibility of metaphysical assumptions, such as whether causality should be regarded as residing in the field vs. in the object.

(6) Social organization and social practice can directly influence the development and use of cognitive processes such as dialectical vs. logical ones.

Nisbett et al. [4] conclude that tools of thought embody a culture's intellectual history, that tools have theories build into them, and that users accept these theories, albeit unknowingly, when they use these tools.

Heine [6] puts the matter as follows:

"Cultural psychology does not view culture as a superficial wrapping of the self, or as a framework within which selves interact, but as something that is intrinsic to the self. It assumes that without culture there is no self, only a biological entity deprived of its potential... Cultural psychology maintains that the process of becoming a self is contingent on individuals interacting with and seizing meanings from the cultural environment..."

Clearly, culture must have an intimate relation with the cognitive functioning of the organizations in which individual humans are embedded and with which they are synergistic in an apparent evolutionary exaptation of individual consciousness (e.g. [7]).

The scientific study of individual consciousness has again become popular, after nearly a century of silence enforced by ideological diktat – the 'dark night of behaviorism' – and Baars' Global Workspace Theory (GWT), [1,8] has emerged as the first among equals in the Darwinian competition between theoretical approaches (e.g. [9]). Other viable viewpoints have, in general, branched off from this seminal line of work. Even Maia and Cleeremans [10], for example, who use connectionist models, state that

"The main difference between our perspective and that of Dehaene, Baars, and their [other global workspace] collaborators, is that they take the brain to consist of specialized modular processes, whereas we believe that computation is more distributed and interactive at a global scale... [T]he existence of massive recurrent connections at all levels of the cortex makes the existence of strongly encapsulated modules... unlikely. In any case, this may simply be a matter of emphasis, as Dehaene et al. suggest that 'global workspace neurons' are widely distributed..."

Wallace and colleagues [11-15] have developed the first comprehensive mathematical model of GWT and many of its possible variants, using a Dretske-like information theory formalism [16-19], extended by techniques from statistical physics, the Large Deviations Program of applied probability, and the topological theory of highly parallel computation. The 'necessary conditions' arguments based on application of the Rate Distortion and Shannon-McMillan Theorems to models of individual cognitive process can, we will show in some detail, be extended in a canonical fashion to institutional cognition of various orders. One particular advance is invocation of a 'broken groupoid' formalism which, based on mutual information measures, provides a highly natural means for treating increasing interaction between individual cognitive modules. This finesses debates on strong encapsulation.

Although individual human consciousness has been socially constructed as a great scientific mystery, institutional cognition is, in fact, far more complex and varied, significantly less constrained by biological evolution, and considerably more efficient in many important respects. Hollan et al. [20], expanding on previous work by Hutchins and collaborators (e.g. [21]), describe these matters in terms of a distributed cognition paradigm:

"The theory of distributed cognition, like any cognitive theory, seeks to understand the organization of cognitive systems. Unlike traditional theories, however, it extends the reach of what is considered *cognitive *beyond the individual to encompass interactions between people and with resources and materials in the environment. It is important from the outset to understand that distributed cognition refers to a perspective on all of cognition, rather than a particular kind of cognition... Distributed cognition looks for cognitive processes, wherever they may occur, on the basis of the functional relationships of elements that participate together in the process. A process is not cognitive simply because it happens in a brain, nor is a process noncognitive simply because it happens in the interactions between many brains... In distributed cognition one expects to find a system that can dynamically configure itself to bring subsystems into coordination to accomplish various functions. A cognitive process is delimited by the functional relationships among the elements that participate in it, rather than by the spatial colocation of the elements... Whereas traditional views look for cognitive events in the manipulation of symbols inside individual actors, distributed cognition looks for a broader class of cognitive events and does not expect all such events to be encompasses by the skin or skull of an individual...

-Cognitive processes may be distributed across the members of a social group.

-Cognitive processes may involve coordination between internal and external (material or environmental) structure.

-Processes may be distributed through time in such a way that the products of earlier events can transform the nature of later events."

Our approach revolves around a 'dual information source', a kind of quasi-language, which is to be associated with certain classes of cognitive process, however these may be instantiated – within or between individuals, or related to systems involving individuals, groups, and their various cultural artifacts.

The ability to engage in culturally-sculpted and enabled organizational cognition, in fact, may be as fundamental to human survival as individual consciousness, which appears to be a very ancient evolutionary adaptation. The dual heritage systems of genes and culture serve at both individual and collective scales of human endeavor [3].

According to the cultural anthropologists, the structures, functions, and innate character of organizational behavior are greatly variable and highly adaptable across social and physical geography, and across history. Individual human consciousness, by contrast, although profoundly shaped by, and indeed synergistic with, culture, remains constrained by the primary biological necessity of single-tasking, leading to the striking phenomenon of inattentional blindness (IAB) when the Rate Distortion Manifold of consciousness become necessarily focused on one primary process to the virtual exclusion of others which might be expected to intrude (e.g. [14,22-24]).

Simons and Chabris [25] detail a particularly spectacular example of IAB. A videotape was made of a basketball game between teams in white and black jerseys. Experimental subjects who viewed the tape were asked to keep silent mental counts of either the total number of passes made by one or the other of the teams, or separate counts of the number of bounce and areal passes. During the game, a figure in a full gorilla suit appears, faces the camera, beats its breast, and walks off the court. About one half of the experimental subjects completely failed to notice the Gorilla during the experiment. See [26] for an extended discussion, and [27] for more recent experiments.

Other case histories, involving an aircraft crew which became fixated on an unexpectedly flashing control panel light during a landing, or a man walking a railroad track while having a cell phone conversation, are less benign.

Generalizing a second order treatment of Baars' Global Workspace model of individual consciousness to organizational structures will suggest the possibility of an analogous collective multitasking, effectively an institutional collective consciousness far more complex than the individual case. There will emerge, however, an institutional analog to individual inattentional blindness, and additional failure modes specific to the complication of communication between multiple workspaces, as well as those related to the failure of individual workspaces within the organization, and to pathological 'lock-in'. Remediation appears severely limited by the effects on it of the externalities so often responsible for the failures themselves.

We begin with an outline of recent work on individual consciousness as a kind of second order iteration of simple cognition, and then make the extensions needed to describe institutional multiple workspaces and their failure modes.

## Formal theory

### 1. The Global Workspace model of individual consciousness

The central ideas of Baars' Global Workspace Theory of individual consciousness are as follows [28]:

(1) The brain can be viewed as a collection of distributed specialized networks (processors).

(2) Consciousness is associated with a global workspace in the brain – a fleeting memory capacity whose focal contents are widely distributed (broadcast) to many unconscious specialized networks.

(3) Conversely, a global workspace can also serve to integrate many competing and cooperating input networks.

(4) Some unconscious networks, called contexts, shape conscious contents, for example unconscious parietal maps modulate visual feature cells that underlie the perception of color in the ventral stream.

(5) Such contexts work together jointly to constrain conscious events.

(6) Motives and emotions can be viewed as goal contexts.

(7) Executive functions work as hierarchies of goal contexts. 

Although this basic approach has been the central focus of many researchers for two decades, consciousness studies has only recently, in the context of a deluge of empirical results from brain imaging experiments, begun digesting the perspective and preparing to move on.

Theory, however, sadly lags experiment. As Atmanspacher [29] has put it,

"To formulate a serious, clear-cut and transparent formal framework for cognitive neuroscience is a challenge comparable to the early stage of physics four centuries ago."

Currently popular agent-based and artificial neural network (ANN) treatments of cognition, consciousness and other higher order mental functions, to take Krebs' view, [30] are little more than sufficiency arguments, in the same sense that a Fourier series expansion can be empirically fitted to nearly any function over a fixed interval without providing real understanding of the underlying structure. Necessary conditions, as Dretske argues [16-19], give considerably more insight.

Wallace [11-14] in effect addresses Baars' theme from Dretske's viewpoint, examining the necessary conditions which the asymptotic limit theorems of information theory impose on the Global Workspace or any similar broadcast system. A central outcome of that work is the incorporation, in a natural manner, of constraints on individual consciousness, i.e. what Baars calls contexts. A particular concern of this work, however, is with the surprisingly wide spectrum of mechanisms which can potentially broadcast focal contents.

The extension to institutional collective consciousness requires examining how cognitive modules can multitask, engaging in more than one global broadcast at the same time, which normal individual human consciousness does not do. The obvious tradeoff, of course, is the very rapid flow of individual consciousness, a matter of a few hundred milliseconds, as opposed to the much slower, if considerably more comprehensive, operations of institutional generalizations.

### 2. Cognition as an information source

Cognition is not consciousness (or institutional collective consciousness, as we will define it). Most mental, many physiological, and a plethora of institutional, functions, while cognitive in a formal sense, hardly ever become entrained into the global broadcast of individual consciousness (or, as we shall see, the many such systems of institutional collective consciousness): one seldom is able to consciously regulate immune function, blood pressure, or the details of binocular tracking and bipedal motion, except to decide 'what shall I look at', 'where shall I walk'. Nonetheless, many individual cognitive processes, conscious or unconscious, appear intimately related to language, broadly speaking. The construction is fairly straightforward [11-14,31].

Atlan and Cohen [2] and Cohen [32] argue, in the context of immune cognition, that the essence of cognitive function involves comparison of a perceived signal with an internal, learned picture of the world, and then, upon that comparison, choice of one response from a much larger repertoire of possible responses.

More formally, an incoming, highly structured, sensory signal is mixed in an unspecified but systematic algorithmic manner with a structured pattern of internal ongoing activity to create a combined path, *x *= (*a*_0_, *a*_1_, ..., *a*_*n*_, ...). Each *a*_*k *_thus represents some functional composition of internal and external messages. Wallace [11] provides two neural network examples.

The combined path *x *is then fed into a highly nonlinear, but otherwise similarly unspecified, decision oscillator, *h*, which generates an output *h*(*x*) that is an element of one of two disjoint sets *B*_0 _and *B*_1 _of possible responses.

Let

*B*_0 _≡ *b*_0_, ..., *b*_*k*_,

*B*_1 _≡ *b*_*k*+1_, ..., *b*_*m*_.

If

*h*(*x*) ∈ *B*_0_,

the pattern is not recognized, and no action is taken. If

*h*(*x*) ∈ *B*_1_,

the pattern is recognized, and some action *b*_*j*_, *k *+ 1 ≤ *j *≤ *m *takes place.

The principal objects of formal interest are paths *x *which trigger pattern recognition-and-response. That is, given a fixed initial state *a*_0_, we examine all possible subsequent paths *x *beginning with *a*_0 _and leading to the event *h*(*x*) ∈ *B*_1_. Thus *h*(*a*_0_, ..., *a*_*j*_) ∈ *B*_0 _for all 0 <*j *<*m*, but *h*(*a*_0_, ..., *a*_*m*_) ∈ *B*_1_.

For each positive integer *n*, let *N*(*n*) be the number of high probability grammatical and syntactical paths of length *n *which begin with some particular *a*_0 _and lead to the condition *h*(*x*) ∈ *B*_1_. Call such paths 'meaningful', assuming, not unreasonably, that *N*(*n*) will be considerably less than the number of all possible paths of length *n *leading from *a*_0 _to the condition *h*(*x*) ∈ *B*_1_.

While the combining algorithm generating the *a*_*i*_, the form of the nonlinear oscillator, and the details of grammar and syntax, are all unspecified in this model, the critical assumption which permits inference on necessary conditions constrained by the asymptotic limit theorems of information theory is that the finite limit

H≡lim⁡n→∞log⁡[N(n)]n     (1)
 MathType@MTEF@5@5@+=feaafiart1ev1aaatCvAUfKttLearuWrP9MDH5MBPbIqV92AaeXatLxBI9gBaebbnrfifHhDYfgasaacH8akY=wiFfYdH8Gipec8Eeeu0xXdbba9frFj0=OqFfea0dXdd9vqai=hGuQ8kuc9pgc9s8qqaq=dirpe0xb9q8qiLsFr0=vr0=vr0dc8meaabaqaciaacaGaaeqabaqabeGadaaakeaacqWGibascqGHHjIUdaWfqaqaaiGbcYgaSjabcMgaPjabc2gaTbWcbaGaemOBa4MaeyOKH4QaeyOhIukabeaakmaalaaabaGagiiBaWMaei4Ba8Maei4zaCMaei4waSLaemOta4KaeiikaGIaemOBa4MaeiykaKIaeiyxa0fabaGaemOBa4gaaiaaxMaacaWLjaWaaeWaaeaacqaIXaqmaiaawIcacaGLPaaaaaa@48BE@

both exists and is independent of the path *x*.

We call such a pattern recognition-and-response cognitive process *ergodic*, whether it occurs within an individual or an institution. Not all cognitive processes are likely to be ergodic in this sense, implying that *H*, if it indeed exists at all, is path dependent, although extension to nearly ergodic processes, in a certain sense, seems possible [11].

Invoking the spirit of the Shannon-McMillan Theorem, essentially the zero-error limit of the Rate Distortion Theorem discussed in the Mathematical Appendix, allows definition of an adiabatically, piecewise stationary, ergodic information source (APSE) **X **associated with stochastic variates *X*_*j *_having joint and conditional probabilities *P*(*a*_0_, ..., *a*_*n*_) and *P*(*a*_*n*_|*a*_0_, ..., *a*_*n*-1_) such that appropriate joint and conditional Shannon uncertainties satisfy the classic relations

H[X]=lim⁡n→∞log⁡[N(n)]n=lim⁡n→∞H(Xn|X0,...,Xn−1)=lim⁡n→∞H(X0,...,Xn)n.
 MathType@MTEF@5@5@+=feaafiart1ev1aaatCvAUfKttLearuWrP9MDH5MBPbIqV92AaeXatLxBI9gBaebbnrfifHhDYfgasaacH8akY=wiFfYdH8Gipec8Eeeu0xXdbba9frFj0=OqFfea0dXdd9vqai=hGuQ8kuc9pgc9s8qqaq=dirpe0xb9q8qiLsFr0=vr0=vr0dc8meaabaqaciaacaGaaeqabaqabeGadaaakeaafaqabeWabaaabaGaemisaGKaei4waSfcbeGae8hwaGLaeiyxa0Laeyypa0ZaaCbeaeaacyGGSbaBcqGGPbqAcqGGTbqBaSqaaiabd6gaUjabgkziUkabg6HiLcqabaGcdaWcaaqaaiGbcYgaSjabc+gaVjabcEgaNjabcUfaBjabd6eaojabcIcaOiabd6gaUjabcMcaPiabc2faDbqaaiabd6gaUbaacqGH9aqpaeaadaWfqaqaaiGbcYgaSjabcMgaPjabc2gaTbWcbaGaemOBa4MaeyOKH4QaeyOhIukabeaakiabdIeaijabcIcaOiabdIfaynaaBaaaleaacqWGUbGBaeqaaOGaeiiFaWNaemiwaG1aaSbaaSqaaiabicdaWaqabaGccqGGSaalcqGGUaGlcqGGUaGlcqGGUaGlcqGGSaalcqWGybawdaWgaaWcbaGaemOBa4MaeyOeI0IaeGymaedabeaakiabcMcaPiabg2da9aqaamaaxababaGagiiBaWMaeiyAaKMaeiyBa0galeaacqWGUbGBcqGHsgIRcqGHEisPaeqaaOWaaSaaaeaacqWGibascqGGOaakcqWGybawdaWgaaWcbaGaeGimaadabeaakiabcYcaSiabc6caUiabc6caUiabc6caUiabcYcaSiabdIfaynaaBaaaleaacqWGUbGBaeqaaOGaeiykaKcabaGaemOBa4gaaiabc6caUaaaaaa@7DBA@

This APSE information source is defined as *dual *to the underlying ergodic cognitive process [11].

The essence of 'adiabatic' is that, when the information source is parametized according to some appropriate scheme, within continuous 'pieces' of that parametization, changes in parameters take place slowly enough so that the information source remains as close to stationary and ergodic as is needed to make the fundamental theorems work. By 'stationary' we mean that probabilities do not change in time, and by 'ergodic' (roughly) that cross-sectional means converge to long-time averages. Between 'pieces' one invokes various kinds of phase change formalism, for example renormalization theory in cases where a mean field approximation is appropriate [11]. Here we will take a somewhat different approach.

Again, not all cognitive processes are likely to have such a dual source, in this formal sense, and the theory is restricted to those which do.

Recall that the Shannon uncertainties *H*(...) are cross-sectional law-of-large-numbers sums of the form - ∑_*k *_*P*_*k *_log[*P*_*k*_], where the *P*_*k *_constitute a probability distribution. See [33-35] for the standard details.

### 3. The cognitive modular network symmetry groupoid

A formal equivalence class algebra can be constructed by choosing different origins *a*_0 _and defining equivalence by the existence of a high probability meaningful path connecting other states to those origins. Disjoint partition by equivalence class, analogous to orbit equivalence classes for dynamical systems, defines the vertices of the proposed network of cognitive dual languages. Each vertex then represents a different information source dual to a cognitive process. This is not a representation of a neural network as such, or of some circuit in silicon. It is, rather, an abstract set of information sources dual to the cognitive processes instantiated by either biological wetware, social process, or their hybrids with each other and with electronic or other cultural artifacts.

**Figure 1:**
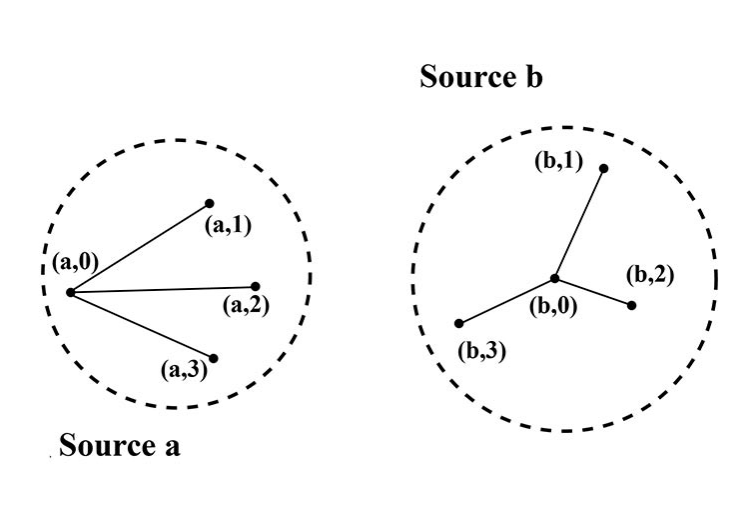
Two (disjoint) equivalence classes of states defined through connection by meaningful paths with different base points, (*a*, 0), (*b*, 0).

This structure generates a groupoid, in the sense of Weinstein [36]. States *a*_*j*_, *a*_*k *_in a set *A *are related by the groupoid morphism if and only if there exists a high probability grammatical path connecting them to the same origin *a*_0_, and tuning across the various possible ways in which that can happen – the different cognitive languages – parametizes the set of equivalence relations and creates the groupoid. See figure 1. This assertion requires some development.

Note that not all possible pairs of states (*a*_*j*_, *a*_*k*_) can be connected by such a morphism, i.e. by a high probability, grammatical and syntactical cognitive path having some particular origin, but those that can define the groupoid element, a morphism *g *= (*a*_*j*_, *a*_*k*_) having the natural inverse *g*^-1 ^= (*a*_*k*_, *a*_*j*_). Given such a pairing, connection by a meaningful path to an origin, it is possible to define 'natural' end-point maps *α*(*g*) = *a*_*j*_, *β*(*g*) = *a*_*k *_from the set of morphisms *G *into *A*, and a formally associative product in the groupoid *g*_1_*g*_2 _provided *α*(*g*_1_*g*_2_) = *α*(*g*_1_), *β*(*g*_1_*g*_2_) = *β*(*g*_2_), and *β*(*g*_1_) = *α*(*g*_2_). Then the product is defined, and associative, i.e. (*g*_1_*g*_2_)*g*_3 _= *g*_1_(*g*_2_*g*_3_).

In addition there are natural left and right identity elements *λ*_*g*_, *ρ*_*g *_such that *λ*_*g*_*g *= *g *= *gρ*_*g *_[36].

An orbit of the groupoid *G *over *A *is an equivalence class for the relation *a*_*j *_~ *Ga*_*k *_if and only if there is a groupoid element *g *with *α*(*g*) = *a*_*j *_and *β*(*g*)= *a*_*k*_.

The isotopy group of *a *∈ *X *consists of those *g *in *G *with *α*(*g*) = *a *= *β*(*g*).

In essence a groupoid is a category in which all morphisms have an inverse, here defined in terms of connection by a meaningful path of an information source dual to a cognitive process.

If *G *is any groupoid over *A*, the map (*α*, *β*) : *G *→ *A *× *A *is a morphism from *G *to the pair groupoid of *A*. The image of (*α*, *β*) is the orbit equivalence relation ~ *G*, and the functional kernel is the union of the isotropy groups. If *f *: *X *→ *Y *is a function, then the kernel of *f*, *ker*(*f*) = [(*x*_1_, *x*_2_) ∈ *X *× *X *: *f *= *f*(*x*_1_) = *f*(*x*_2_)] defines an equivalence relation.

As Weinstein (1996) points out, the morphism (*α*, *β*) suggests another way of looking at groupoids. A groupoid over *A *identifies not only which elements of *A *are equivalent to one another (isomorphic), but *it also parametizes the different ways (isomorphisms) in which two elements can be equivalent*, i.e. all possible information sources dual to some set of cognitive processes. Given the information theoretic characterization of cognition presented above, this produces a full modular cognitive network in a highly natural manner.

Brown [37] describes the fundamental structure as follows:

"A groupoid should be thought of as a group with many objects, or with many identities... A groupoid with one object is essentially just a group. So the notion of groupoid is an extension of that of groups. It gives an additional convenience, flexibility and range of applications...

EXAMPLE 1. A disjoint union [of groups] *G *= ∪_*λ*_*G*_*λ*_, *λ *∈ Λ is a groupoid: the product *ab *is defined if and only if *a*, *b *belong to the same *G*_*λ*_, and *ab *is then just the product in the group *G*_*λ*_.

There is an identity 1_*λ *_for each *λ *∈ Λ. The maps *α*, *β *coincide and map *G*_*λ *_to *λ*, *λ *∈ Λ.

EXAMPLE 2. An equivalence relation *R *on [a set] *X *becomes a groupoid with *α*, *β *: *R *→ *X *the two projections, and product (*x*, *y*)(*y*, *z*) = (*x*, *z*) whenever (*x*, *y*), (*y*, *z*) ∈ *R*. There is an identity, namely (*x*, *x*), for each *x *∈ *X*..."

Weinstein [36] makes the following fundamental point:

"Almost every interesting equivalence relation on a space *B *arises in a natural way as the orbit equivalence relation of some groupoid *G *over *B*. Instead of dealing directly with the orbit space *B*/*G *as an object in the category *S*_*map *_of sets and mappings, one should consider instead the groupoid *G *itself as an object in the category *G*_*htp *_of groupoids and homotopy classes of morphisms."

Later we will explore homotopy in paths generated by information sources.

The groupoid approach has become quite popular in the study of networks of coupled dynamical systems which can be defined by differential equation models, (e.g. [38-40]). Here we have outlined how to extend the technique to networks of interacting information sources which, in a dual sense, characterize cognitive processes, and cannot at all be described by the usual differential equation models. These latter, it seems, are much the spiritual offspring of 18th Century mechanical clocks. Cognitive and conscious processes in humans involve neither computers nor clocks, but remain constrained by the limit theorems of information theory, and these permit scientific inference on necessary conditions.

### 4. Internal forces breaking the symmetry groupoid

The symmetry groupoid for cognitive modules is generated by the possible ways in which states *a*_*j*_, *a*_*k *_can be connected to some particular origin by a meaningful path of an information source dual to a cognitive process. These are different, and in this approximation, non-interacting cognitive processes. But symmetry groupoids, like symmetry groups, are made to be broken: by internal cross-talk akin to spin-orbit interactions within a symmetric atom, and by cross-talk with slower, external, information sources, akin to putting a symmetric atom in a powerful magnetic or electric field.

**Figure 2:**
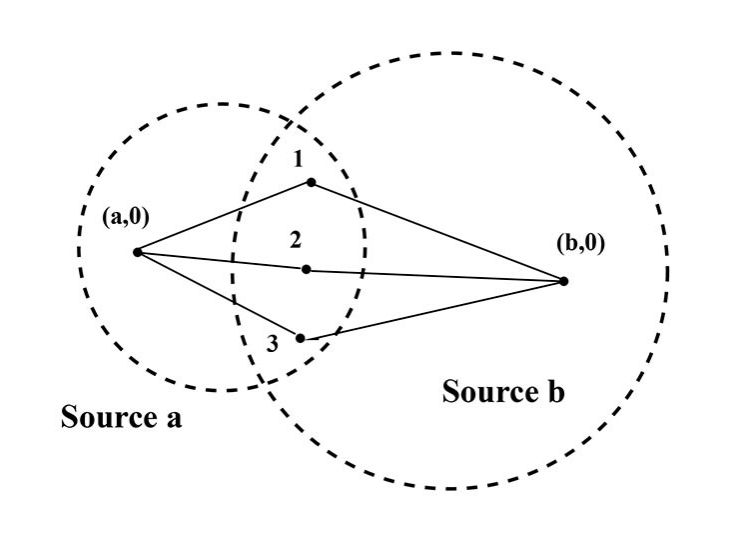
Complications due to crosstalk between information sources: States labeled 1, 2, 3 can be connected by meaningful paths with two different base points, (*a*, 0), (*b*, 0). Defining equivalence classes becomes much more difficult.

Figure 2 illustrates the problem, in which the states labeled 1, 2, 3 are connected to two different base points, written as (*a*, 0), (*b*, 0).

First suppose that linkages can fleetingly occur between the ordinarily disjoint cognitive modules defined by the network groupoid. In the spirit of [11], this is represented by establishment of a non-zero mutual information measure between them: a cross-talk which breaks the strict groupoid symmetry developed above.

Wallace [11] describes this structure in terms of fixed magnitude disjunctive strong ties which give the equivalence class partitioning of modules, and nondisjunctive weak ties which link modules across the partition, and parametizes the overall structure by the average strength of the weak ties, to use Granovetter's [41] term. A different approach, [12], outlined here, is to simply look at the average number of fixed-strength nondisjunctive links in a random topology. These are obviously just two analytically tractable limits of a much more complicated regime of possibilities. This circumstance, in fact, suggests the operation of selection pressures both in the evolution of individual consciousness and in the Lamarckian evolution of institutions, matters discussed elsewhere [7].

Since we know nothing about how the cross-talk connections can occur, we – at first – construct a simple random graph in the classic Erdos/Renyi manner. Suppose there are *M *disjoint cognitive modules – *M *elements of the equivalence class algebra of languages dual to some cognitive process – which we now take to be the vertices of a possible graph.

For *M *very large, following [42], when edges (defined by establishment of a fixed-strength mutual information measure between the graph vertices) are added at random to *M *initially disconnected vertices, a remarkable transition occurs when the number of edges becomes approximately *M*/2. Erdos and Renyi [43] studied random graphs with *M *vertices and (*M*/2)(1 + *μ*) edges as *M *→ ∞, and discovered that such graphs almost surely have the following properties [44-49]:

[1] If *μ *< 0, only small trees and unicyclic components are present, where a unicyclic component is a tree with one additional edge; moreover, the size of the largest tree component is (*μ *- ln(1 + *μ*))^-1 ^+ O
 MathType@MTEF@5@5@+=feaafiart1ev1aaatCvAUfKttLearuWrP9MDH5MBPbIqV92AaeXatLxBI9gBaebbnrfifHhDYfgasaacH8akY=wiFfYdH8Gipec8Eeeu0xXdbba9frFj0=OqFfea0dXdd9vqai=hGuQ8kuc9pgc9s8qqaq=dirpe0xb9q8qiLsFr0=vr0=vr0dc8meaabaqaciaacaGaaeqabaqabeGadaaakeaat0uy0HwzTfgDPnwy1egaryqtHrhAL1wy0L2yHvdaiqaacqWFoe=taaa@383C@(log log *n*).

[2] If *μ *= 0, however, the largest component has size of order *M*^2/3^.

[3] If *μ *> 0, there is a unique giant component (GC) whose size is of order *M*; in fact, the size of this component is asymptotically *αM*, where *μ *= -*α*^-1 ^[ln(1 - *α*) - 1], which has an explicit solution for *α *in terms of the Lambert W-function. Thus, for example, a random graph with approximately *M *ln(2) edges will have a giant component containing ≈ *M*/2 vertices.

Such a phase transition initiates a new, collective, cognitive phenomenon. At the level of the individual mind, unconscious cognitive modules link up to become the General Broadcast associated with consciousness, emergently defined by a set of cross-talk mutual information measures between interacting unconscious cognitive submodules. The source uncertainty, *H*, of the language dual to the collective cognitive process, which characterizes the richness of the cognitive language of the workspace, will grow as some monotonic function of the size of the GC, as more and more unconscious processes are incorporated into it. Wallace [11] provides details.

Others have taken similar network phase transition approaches to assemblies of neurons, e.g. neuropercolation [50,51], but their work has not focused explicitly on modular networks of cognitive processes, which may or may not be instantiated by neurons. Restricting analysis to such modular networks finesses much of the underlying conceptual difficulty, and permits use of the asymptotic limit theorems of information theory and the import of techniques from statistical physics, a matter we will discuss later.

Again, this is only one limit in a continuum of possible models. Another limiting case involves a mean field approximation which examines changes in the average strength of coupling, rather than the average number of links [11].

### 5. External forces breaking the symmetry groupoid

Just as a higher order information source, associated with the GC of a random or semirandom graph, can be constructed out of the interlinking of unconscious cognitive modules by mutual information, so too external information sources, for example in humans the cognitive immune and other physiological systems, and embedding sociocultural structures, can be represented as slower-acting information sources whose influence on the GC can be felt in a collective mutual information measure. For machines or institutions these would be the onion-like 'structured environment', to be viewed as among Baars' contexts [1,8,28]. The collective mutual information measure will, through the Joint Asymptotic Equipartition Theorem which generalizes the Shannon-McMillan Theorem, be the splitting criterion for high and low probability joint paths across the entire system.

The tool for this is network information theory ([35], p. 388). Given three interacting information sources, *Y*_1_, *Y*_2_, *Z*, the splitting criterion, taking *Z *as the 'external context', is given by

*I*(*Y*_1_, *Y*_2_|*Z*) = *H*(*Z*) + *H*(*Y*_1_|*Z*) + *H*(*Y*_2_|*Z*) - *H*(*Y*_1_, *Y*_2_, *Z*),     (2)

where *H*(..|..) and *H*(..,..,..) represent conditional and joint uncertainties [33-35].

This generalizes to

I(Y1,...Yn|Z)=H(Z)+∑j=1nH(Yj|Z)−H(Y1,...,Yn,Z).     (3)
 MathType@MTEF@5@5@+=feaafiart1ev1aaatCvAUfKttLearuWrP9MDH5MBPbIqV92AaeXatLxBI9gBaebbnrfifHhDYfgasaacH8akY=wiFfYdH8Gipec8Eeeu0xXdbba9frFj0=OqFfea0dXdd9vqai=hGuQ8kuc9pgc9s8qqaq=dirpe0xb9q8qiLsFr0=vr0=vr0dc8meaabaqaciaacaGaaeqabaqabeGadaaakeaacqWGjbqscqGGOaakcqWGzbqwdaWgaaWcbaGaeGymaedabeaakiabcYcaSiabc6caUiabc6caUiabc6caUiabdMfaznaaBaaaleaacqWGUbGBaeqaaOGaeiiFaWNaemOwaOLaeiykaKIaeyypa0JaemisaGKaeiikaGIaemOwaOLaeiykaKIaey4kaSYaaabCaeaacqWGibascqGGOaakcqWGzbqwdaWgaaWcbaGaemOAaOgabeaakiabcYha8jabdQfaAjabcMcaPiabgkHiTiabdIeaijabcIcaOiabdMfaznaaBaaaleaacqaIXaqmaeqaaOGaeiilaWIaeiOla4IaeiOla4IaeiOla4IaeiilaWIaemywaK1aaSbaaSqaaiabd6gaUbqabaGccqGGSaalcqWGAbGwcqGGPaqkcqGGUaGlaSqaaiabdQgaQjabg2da9iabigdaXaqaaiabd6gaUbqdcqGHris5aOGaaCzcaiaaxMaadaqadaqaaiabiodaZaGaayjkaiaawMcaaaaa@6460@

If we assume the General Broadcast/Giant Component to involve a very rapidly shifting, and indeed highly tunable, dual information source *X*, embedding contextual cognitive modules like the immune system will have a set of significantly slower-responding sources *Y*_*j*_, *j *= 1..*m*, and external social, cultural and other environmental processes will be characterized by even more slowly-acting sources *Z*_*k*_, *k *= 1..*n*. Mathematical induction on equation (3) gives a complicated expression for a mutual information splitting criterion which we write as

*I*(*X*|*Y*_1_, .., *Y*_*m*_|*Z*_1_, .., *Z*_*n*_).     (4)

This encompasses, at the individual level, a fully interpenetrating biopsychosociocultural structure for consciousness, one in which Baars' contexts act as important, but flexible, boundary conditions, defining the underlying topology available to the far more rapidly shifting global workspace [11-14].

This result does not commit the mereological fallacy, of which Bennett and Hacker [52] accuse the many excessively neurocentric perspectives on consciousness in humans, that is, the mistake of imputing to a part of a system the characteristics which require functional entirety. The underlying concept of this fallacy should extend to machines or organizations interacting with their environments.

The central argument of this paper is to generalize this result to the institutional level, albeit operating on a time scale much slower than individual consciousness.

### 6. Punctuation phenomena

As many have noted – see [11-14] for more discussion – equation (1),

H≡lim⁡n→∞log⁡[N(n)]n,
 MathType@MTEF@5@5@+=feaafiart1ev1aaatCvAUfKttLearuWrP9MDH5MBPbIqV92AaeXatLxBI9gBaebbnrfifHhDYfgasaacH8akY=wiFfYdH8Gipec8Eeeu0xXdbba9frFj0=OqFfea0dXdd9vqai=hGuQ8kuc9pgc9s8qqaq=dirpe0xb9q8qiLsFr0=vr0=vr0dc8meaabaqaciaacaGaaeqabaqabeGadaaakeaacqWGibascqGHHjIUdaWfqaqaaiGbcYgaSjabcMgaPjabc2gaTbWcbaGaemOBa4MaeyOKH4QaeyOhIukabeaakmaalaaabaGagiiBaWMaei4Ba8Maei4zaCMaei4waSLaemOta4KaeiikaGIaemOBa4MaeiykaKIaeiyxa0fabaGaemOBa4gaaiabcYcaSaaa@45E1@

is homologous to the thermodynamic limit in the definition of the free energy density of a physical system. This has the form

F(K)≡lim⁡V→∞log⁡[Z(K)]V,     (5)
 MathType@MTEF@5@5@+=feaafiart1ev1aaatCvAUfKttLearuWrP9MDH5MBPbIqV92AaeXatLxBI9gBaebbnrfifHhDYfgasaacH8akY=wiFfYdH8Gipec8Eeeu0xXdbba9frFj0=OqFfea0dXdd9vqai=hGuQ8kuc9pgc9s8qqaq=dirpe0xb9q8qiLsFr0=vr0=vr0dc8meaabaqaciaacaGaaeqabaqabeGadaaakeaacqWGgbGrcqGGOaakcqWGlbWscqGGPaqkcqGHHjIUdaWfqaqaaiGbcYgaSjabcMgaPjabc2gaTbWcbaGaemOvayLaeyOKH4QaeyOhIukabeaakmaalaaabaGagiiBaWMaei4Ba8Maei4zaCMaei4waSLaemOwaOLaeiikaGIaem4saSKaeiykaKIaeiyxa0fabaGaemOvayfaaiabcYcaSiaaxMaacaWLjaWaaeWaaeaacqaI1aqnaiaawIcacaGLPaaaaaa@4BE5@

where *F *is the free energy density, *K *the inverse temperature, *V *the system volume, and *Z*(*K*) is the partition function defined by the system Hamiltonian.

Wallace [11] shows at some length how this homology permits the natural transfer of renormalization methods from statistical mechanics to information theory. In the spirit of the Large Deviations Program of applied probability theory, this produces phase transitions and analogs to evolutionary punctuation in systems characterized by piecewise, adiabatically stationary, ergodic information sources. These biological phase changes appear to be ubiquitous in natural systems and can be expected to dominate machine and organizational behaviors as well. Wallace [53] uses these arguments to explore the differences and similarities between evolutionary punctuation in genetic and learning plateaus in neural systems.

### 7. Institutional collective consciousness

The random network development above is predicated on there being a variable average number of fixed-strength linkages between components. Clearly, the mutual information measure of cross-talk is not inherently fixed, but can continuously vary in magnitude. This can be addressed by a parametized renormalization. In essence the modular network structure linked by mutual information interactions has a topology depending on the degree of interaction of interest. Suppose we define an interaction parameter *ω*, a real positive number, and look at geometric structures defined in terms of linkages which are zero if mutual information is less than, and 'renormalized' to unity if greater than, *ω*. Any given *ω *will define a regime of giant components of network elements linked by mutual information greater than or equal to it.

*The fundamental conceptual trick at this point is to invert the argument* : A given topology for the giant component will, in turn, define some critical value, *ω*_*C *_so that network elements interacting by mutual information less than that value will be unable to participate, i.e. will be locked out and not be consciously perceived. We hence are assuming that the *ω *is a tunable, syntactically-dependent, detection limit, and depends critically on the instantaneous topology of the giant component defining, for the human mind, the general broadcast of consciousness. That topology is, fundamentally, the basic tunable syntactic filter across the underlying modular symmetry groupoid, and variation in *ω *is only one aspect of a much more general topological shift. More detailed analysis is given below in terms of a topological rate distortion manifold.

There is considerable empirical evidence from fMRI brain imaging experiments to show that individual human consciousness involves a single global component, a matter leading necessarily to the phenomenon of inattentional blindness [14]. Cognitive submodules within institutions – individuals, departments, formal and informal workgroups – by contrast, can do more than one thing, and indeed, are usually required to multitask. Clearly this will lessen the probability of inattentional blindness, but does not eliminate it, and introduces other failure modes.

We must, for organizations as opposed to individual minds, postulate a set of crosstalk information measures between cognitive submodules, each associated with its own giant component having its own special topology.

Suppose the set of giant components at some 'time' *k *is characterized by a set of parameters Ω_*k *_≡ ω1k
 MathType@MTEF@5@5@+=feaafiart1ev1aaatCvAUfKttLearuWrP9MDH5MBPbIqV92AaeXatLxBI9gBaebbnrfifHhDYfgasaacH8akY=wiFfYdH8Gipec8Eeeu0xXdbba9frFj0=OqFfea0dXdd9vqai=hGuQ8kuc9pgc9s8qqaq=dirpe0xb9q8qiLsFr0=vr0=vr0dc8meaabaqaciaacaGaaeqabaqabeGadaaakeaaiiGacqWFjpWDdaqhaaWcbaGaeGymaedabaGaem4AaSgaaaaa@30FC@, ..., ωmk
 MathType@MTEF@5@5@+=feaafiart1ev1aaatCvAUfKttLearuWrP9MDH5MBPbIqV92AaeXatLxBI9gBaebbnrfifHhDYfgasaacH8akY=wiFfYdH8Gipec8Eeeu0xXdbba9frFj0=OqFfea0dXdd9vqai=hGuQ8kuc9pgc9s8qqaq=dirpe0xb9q8qiLsFr0=vr0=vr0dc8meaabaqaciaacaGaaeqabaqabeGadaaakeaaiiGacqWFjpWDdaqhaaWcbaGaemyBa0gabaGaem4AaSgaaaaa@316F@. Fixed parameter values define a particular giant component set having a particular set of topological structures. Suppose that, over a sequence of 'times' the set of giant components can be characterized by a (possibly coarse-grained) path *x*_*n *_= Ω_0_, Ω_1_, ..., Ω_*n*-1 _having significant serial correlations which, in fact, permit definition of an adiabatically, piecewise stationary, ergodic (APSE) information source in the sense above. Call that information source **X**.

Suppose, again in the manner of [11,14], that a set of (external or internal) signals impinging on the set of giant components, is also highly structured and forms another APSE information source **Y **which interacts not only with the system of interest globally, but specifically with the tuning parameters of the set of giant components characterized by **X**. **Y **is necessarily associated with a set of paths *y*_*n*_.

Pair the two sets of paths into a joint path *z*_*n *_≡ (*x*_*n*_, *y*_*n*_), and invoke some inverse coupling parameter, *K*, between the information sources and their paths. By the arguments of [11] this leads to phase transition punctuation of *I*[*K*], the mutual information between **X **and **Y**, under either the Joint Asymptotic Equipartition Theorem, or, given a distortion measure, under the Rate Distortion Theorem.

*I*[*K*] is a splitting criterion between high and low probability pairs of paths, and partakes of the homology with free energy density described above. Attentional focusing by the institution then itself becomes a punctuated event in response to increasing linkage between the organization and an external structured signal, or some particular system of internal events. This iterated argument parallels the extension of the General Linear Model into the Hierarchical Linear Model of regression theory.

Call this the Hierarchical Cognitive Model (HCM). For individual consciousness, there is only one giant component. For an institution, there will be a larger, and often very large, set of them.

This leads to the possibility of new failure modes related to impaired communication between Giant Components.

That is, a complication specific to high order institutional cognition lies in the necessity of information transfer between giant components. The form and function of such interactions will, of course, be determined by the nature of the particular institution, but, synchronous or asynchronous, contact between giant components is circumscribed by the Rate Distortion Theorem. That theorem, reviewed in the Mathematical Appendix, states that, for a given maximum acceptable critical average distortion, there is a limiting maximum information transmission rate, such that messages sent at less than that limit are guaranteed to have average distortion less than the critical maximum. Too rapid transmission between parallel global workspaces – information overload – violates that condition, and guarantees large average distortion. This is a likely failure mode which appears unique to multiple workspace systems which, if the workspaces are sufficiently numerous, diverse, and able to communicate accurately with each other, may otherwise have a lessened probability of inattentional blindness.

Other failure modes will become apparent in due course.

### 8. The dynamical groupoid

A fundamental homology between the information source uncertainty dual to a cognitive process and the free energy density of a physical system arises, in part, from the formal similarity between their definitions in the asymptotic limit. Information source uncertainty can be defined as in equation (1). This is quite analogous to the free energy density of a physical system, equation (5).

Feynman [54] provides a series of physical examples, based on Bennett's work, where this homology is, in fact, an identity, at least for very simple systems. Bennett argues, in terms of idealized irreducibly elementary computing machines, that the information contained in a message can be viewed as the work saved by not needing to recompute what has been transmitted.

Feynman explores in some detail Bennett's ideal microscopic machine designed to extract useful work from a transmitted message. The essential argument is that computing, in any form, takes work. Thus the more complicated a cognitive process, measured by its information source uncertainty, the greater its energy consumption, and our ability to provide energy to the brain is limited: Typically a unit of brain tissue consumes an order of magnitude more energy than a unit of any other tissue. Inattentional blindness emerges as an inevitable thermodynamic limit on processing capacity in a topologically-fixed global workspace, i.e. one which has been strongly configured about a particular task. Institutional generalizations seem obvious.

Understanding the time dynamics of cognitive systems away from phase transition critical points requires a phenomenology similar to the Onsager relations of nonequilibrium thermodynamics. If the dual source uncertainty of a cognitive process is parametized by some vector of quantities **K **≡ (*K*_1_, ..., *K*_*m*_), then, in analogy with nonequilibrium thermodynamics, gradients in the *K*_*j *_of the *disorder*, defined as

S≡H(K)−∑j=1mKj∂H/∂Kj     (6)
 MathType@MTEF@5@5@+=feaafiart1ev1aaatCvAUfKttLearuWrP9MDH5MBPbIqV92AaeXatLxBI9gBaebbnrfifHhDYfgasaacH8akY=wiFfYdH8Gipec8Eeeu0xXdbba9frFj0=OqFfea0dXdd9vqai=hGuQ8kuc9pgc9s8qqaq=dirpe0xb9q8qiLsFr0=vr0=vr0dc8meaabaqaciaacaGaaeqabaqabeGadaaakeaacqWGtbWucqGHHjIUcqWGibascqGGOaakieqacqWFlbWscqGGPaqkcqGHsisldaaeWbqaaiabdUealnaaBaaaleaacqWGQbGAaeqaaOGaeyOaIyRaemisaGKaei4la8IaeyOaIyRaem4saS0aaSbaaSqaaiabdQgaQbqabaaabaGaemOAaOMaeyypa0JaeGymaedabaGaemyBa0ganiabggHiLdGccaWLjaGaaCzcamaabmaabaGaeGOnaydacaGLOaGaayzkaaaaaa@4964@

become of central interest.

Equation (6) is similar to the definition of entropy in terms of the free energy density of a physical system, as suggested by the homology between free energy density and information source uncertainty.

Pursuing the homology further, the generalized Onsager relations defining temporal dynamics become

dKj/dt=∑iLj,i∂S/∂Ki,     (7)
 MathType@MTEF@5@5@+=feaafiart1ev1aaatCvAUfKttLearuWrP9MDH5MBPbIqV92AaeXatLxBI9gBaebbnrfifHhDYfgasaacH8akY=wiFfYdH8Gipec8Eeeu0xXdbba9frFj0=OqFfea0dXdd9vqai=hGuQ8kuc9pgc9s8qqaq=dirpe0xb9q8qiLsFr0=vr0=vr0dc8meaabaqaciaacaGaaeqabaqabeGadaaakeaacqWGKbazcqWGlbWsdaWgaaWcbaGaemOAaOgabeaakiabc+caViabdsgaKjabdsha0jabg2da9maaqafabaGaemitaW0aaSbaaSqaaiabdQgaQjabcYcaSiabdMgaPbqabaGccqGHciITcqWGtbWucqGGVaWlcqGHciITcqWGlbWsdaWgaaWcbaGaemyAaKgabeaaaeaacqWGPbqAaeqaniabggHiLdGccqGGSaalcaWLjaGaaCzcamaabmaabaGaeG4naCdacaGLOaGaayzkaaaaaa@49F9@

where the *L*_*j*, *i *_are, in first order, constants reflecting the nature of the underlying cognitive phenomena. The L-matrix is to be viewed empirically, in the same spirit as the slope and intercept of a regression model, and may have structure far different than familiar from more simple chemical or physical processes. The *∂S*/*∂K *are analogous to thermodynamic forces in a chemical system, and may be subject to override by external physiological driving mechanisms [55]. We will return to this below in terms of analogs to ecosystem resilience.

Equations (6) and (7) can be derived in a simple parameter-free covariant manner which relies on the underlying topology of the information source space implicit to the development. Different cognitive phenomena have, according to our development, dual information sources, and we are interested in the local properties of the system near a particular reference state. We impose a topology on the system, so that, near a particular 'language' *A*, dual to an underlying cognitive process, there is (in some sense) an open set *U *of closely similar languages *Â*, such that *A*, *Â *⊂ *U*. Note that it may be necessary to coarse-grain the system's responses to define these information sources. The problem is to proceed in such a way as to preserve the underlying essential topology, while eliminating 'high frequency noise'. The formal tools for this can be found, e.g., in Chapter 8 of [56].

Since the information sources dual to the cognitive processes are similar, for all pairs of languages *A*, *Â *in *U*, it is possible to:

[1] Create an embedding alphabet which includes all symbols allowed to both of them.

[2] Define an information-theoretic distortion measure in that extended, joint alphabet between any high probability (i.e. grammatical and syntactical) paths in *A *and *Â*, which we write as *d*(*Ax*, *Âx*) [35]. Note that these languages do not interact, in this approximation.

[3] Define a metric on *U*, for example,

ℳ(A,A^)=|lim⁡∫A,A^d(Ax,A^x)∫A,Ad(Ax,Ax^)−1|,     (8)
 MathType@MTEF@5@5@+=feaafiart1ev1aaatCvAUfKttLearuWrP9MDH5MBPbIqV92AaeXatLxBI9gBaebbnrfifHhDYfgasaacH8akY=wiFfYdH8Gipec8Eeeu0xXdbba9frFj0=OqFfea0dXdd9vqai=hGuQ8kuc9pgc9s8qqaq=dirpe0xb9q8qiLsFr0=vr0=vr0dc8meaabaqaciaacaGaaeqabaqabeGadaaakeaat0uy0HwzTfgDPnwy1egaryqtHrhAL1wy0L2yHvdaiqaacqWFZestcqGGOaakcqWGbbqqcqGGSaalcuWGbbqqgaqcaiabcMcaPiabg2da9iabcYha8jGbcYgaSjabcMgaPjabc2gaTnaalaaabaWaa8qeaeaacqWGKbazcqGGOaakcqWGbbqqcqWG4baEcqGGSaalcuWGbbqqgaqcaiabdIha4jabcMcaPaWcbaGaemyqaeKaeiilaWIafmyqaeKbaKaaaeqaniabgUIiYdaakeaadaWdraqaaiabdsgaKjabcIcaOiabdgeabjabdIha4jabcYcaSiabdgeabjqbdIha4zaajaGaeiykaKcaleaacqWGbbqqcqGGSaalcqWGbbqqaeqaniabgUIiYdaaaOGaeyOeI0IaeGymaeJaeiiFaWNaeiilaWIaaCzcaiaaxMaadaqadaqaaiabiIda4aGaayjkaiaawMcaaaaa@671D@

using an appropriate integration limit argument over the high probability paths. Note that the integration in the denominator is over different paths within *A *itself, while in the numerator it is between different paths in *A *and *Â*.

Consideration suggests ℳ
 MathType@MTEF@5@5@+=feaafiart1ev1aaatCvAUfKttLearuWrP9MDH5MBPbIqV92AaeXatLxBI9gBamrtHrhAL1wy0L2yHvtyaeHbnfgDOvwBHrxAJfwnaebbnrfifHhDYfgasaacH8akY=wiFfYdH8Gipec8Eeeu0xXdbba9frFj0=OqFfea0dXdd9vqai=hGuQ8kuc9pgc9s8qqaq=dirpe0xb9q8qiLsFr0=vr0=vr0dc8meaabaqaciaacaGaaeqabaWaaeGaeaaakeaaimaacqWFZestaaa@3790@ is a formal metric, having

ℳ
 MathType@MTEF@5@5@+=feaafiart1ev1aaatCvAUfKttLearuWrP9MDH5MBPbIqV92AaeXatLxBI9gBamrtHrhAL1wy0L2yHvtyaeHbnfgDOvwBHrxAJfwnaebbnrfifHhDYfgasaacH8akY=wiFfYdH8Gipec8Eeeu0xXdbba9frFj0=OqFfea0dXdd9vqai=hGuQ8kuc9pgc9s8qqaq=dirpe0xb9q8qiLsFr0=vr0=vr0dc8meaabaqaciaacaGaaeqabaWaaeGaeaaakeaaimaacqWFZestaaa@3790@(*A*, *B*) ≥ 0, ℳ
 MathType@MTEF@5@5@+=feaafiart1ev1aaatCvAUfKttLearuWrP9MDH5MBPbIqV92AaeXatLxBI9gBamrtHrhAL1wy0L2yHvtyaeHbnfgDOvwBHrxAJfwnaebbnrfifHhDYfgasaacH8akY=wiFfYdH8Gipec8Eeeu0xXdbba9frFj0=OqFfea0dXdd9vqai=hGuQ8kuc9pgc9s8qqaq=dirpe0xb9q8qiLsFr0=vr0=vr0dc8meaabaqaciaacaGaaeqabaWaaeGaeaaakeaaimaacqWFZestaaa@3790@(*A*, *A*) = 0, ℳ
 MathType@MTEF@5@5@+=feaafiart1ev1aaatCvAUfKttLearuWrP9MDH5MBPbIqV92AaeXatLxBI9gBamrtHrhAL1wy0L2yHvtyaeHbnfgDOvwBHrxAJfwnaebbnrfifHhDYfgasaacH8akY=wiFfYdH8Gipec8Eeeu0xXdbba9frFj0=OqFfea0dXdd9vqai=hGuQ8kuc9pgc9s8qqaq=dirpe0xb9q8qiLsFr0=vr0=vr0dc8meaabaqaciaacaGaaeqabaWaaeGaeaaakeaaimaacqWFZestaaa@3790@(*A*, *B*) = ℳ
 MathType@MTEF@5@5@+=feaafiart1ev1aaatCvAUfKttLearuWrP9MDH5MBPbIqV92AaeXatLxBI9gBamrtHrhAL1wy0L2yHvtyaeHbnfgDOvwBHrxAJfwnaebbnrfifHhDYfgasaacH8akY=wiFfYdH8Gipec8Eeeu0xXdbba9frFj0=OqFfea0dXdd9vqai=hGuQ8kuc9pgc9s8qqaq=dirpe0xb9q8qiLsFr0=vr0=vr0dc8meaabaqaciaacaGaaeqabaWaaeGaeaaakeaaimaacqWFZestaaa@3790@(*B*, *A*), ℳ
 MathType@MTEF@5@5@+=feaafiart1ev1aaatCvAUfKttLearuWrP9MDH5MBPbIqV92AaeXatLxBI9gBamrtHrhAL1wy0L2yHvtyaeHbnfgDOvwBHrxAJfwnaebbnrfifHhDYfgasaacH8akY=wiFfYdH8Gipec8Eeeu0xXdbba9frFj0=OqFfea0dXdd9vqai=hGuQ8kuc9pgc9s8qqaq=dirpe0xb9q8qiLsFr0=vr0=vr0dc8meaabaqaciaacaGaaeqabaWaaeGaeaaakeaaimaacqWFZestaaa@3790@(*A*, *C *) ≤ ℳ
 MathType@MTEF@5@5@+=feaafiart1ev1aaatCvAUfKttLearuWrP9MDH5MBPbIqV92AaeXatLxBI9gBamrtHrhAL1wy0L2yHvtyaeHbnfgDOvwBHrxAJfwnaebbnrfifHhDYfgasaacH8akY=wiFfYdH8Gipec8Eeeu0xXdbba9frFj0=OqFfea0dXdd9vqai=hGuQ8kuc9pgc9s8qqaq=dirpe0xb9q8qiLsFr0=vr0=vr0dc8meaabaqaciaacaGaaeqabaWaaeGaeaaakeaaimaacqWFZestaaa@3790@(*A*, *B*) + ℳ
 MathType@MTEF@5@5@+=feaafiart1ev1aaatCvAUfKttLearuWrP9MDH5MBPbIqV92AaeXatLxBI9gBamrtHrhAL1wy0L2yHvtyaeHbnfgDOvwBHrxAJfwnaebbnrfifHhDYfgasaacH8akY=wiFfYdH8Gipec8Eeeu0xXdbba9frFj0=OqFfea0dXdd9vqai=hGuQ8kuc9pgc9s8qqaq=dirpe0xb9q8qiLsFr0=vr0=vr0dc8meaabaqaciaacaGaaeqabaWaaeGaeaaakeaaimaacqWFZestaaa@3790@(*B*, *C*).

Other metrics seem possible on *U*.

Note that these three conditions can be used to define equivalence classes of *languages*, where previously we defined equivalence classes of *states *which could be linked by high probability, grammatical and syntactical, paths. This led to the characterization of different information sources. Here we construct an entity, formally a topological manifold having a metric, which is an equivalence class of information sources. This is, we will show, a classic differentiable manifold. The set of such equivalence classes defines the *dynamical groupoid*, and questions arise regarding mechanisms, internal or external, which can break that groupoid symmetry, as in the previous example.

Indeed, since *H *and ℳ
 MathType@MTEF@5@5@+=feaafiart1ev1aaatCvAUfKttLearuWrP9MDH5MBPbIqV92AaeXatLxBI9gBamrtHrhAL1wy0L2yHvtyaeHbnfgDOvwBHrxAJfwnaebbnrfifHhDYfgasaacH8akY=wiFfYdH8Gipec8Eeeu0xXdbba9frFj0=OqFfea0dXdd9vqai=hGuQ8kuc9pgc9s8qqaq=dirpe0xb9q8qiLsFr0=vr0=vr0dc8meaabaqaciaacaGaaeqabaWaaeGaeaaakeaaimaacqWFZestaaa@3790@ are both scalars, a 'covariant' derivative can be defined directly as

dH/dℳ=lim⁡A^→AH(A)−H(A^)ℳ(A,A^),     (9)
 MathType@MTEF@5@5@+=feaafiart1ev1aaatCvAUfKttLearuWrP9MDH5MBPbIqV92AaeXatLxBI9gBaebbnrfifHhDYfgasaacH8akY=wiFfYdH8Gipec8Eeeu0xXdbba9frFj0=OqFfea0dXdd9vqai=hGuQ8kuc9pgc9s8qqaq=dirpe0xb9q8qiLsFr0=vr0=vr0dc8meaabaqaciaacaGaaeqabaqabeGadaaakeaacqWGKbazcqWGibascqGGVaWlcqWGKbazt0uy0HwzTfgDPnwy1egaryqtHrhAL1wy0L2yHvdaiqaacqWFZestcqGH9aqpdaWfqaqaaiGbcYgaSjabcMgaPjabc2gaTbWcbaGafmyqaeKbaKaacqGHsgIRcqWGbbqqaeqaaOWaaSaaaeaacqWGibascqGGOaakcqWGbbqqcqGGPaqkcqGHsislcqWGibascqGGOaakcuWGbbqqgaqcaiabcMcaPaqaaiab=ntinjabcIcaOiabdgeabjabcYcaSiqbdgeabzaajaGaeiykaKcaaiabcYcaSiaaxMaacaWLjaWaaeWaaeaacqaI5aqoaiaawIcacaGLPaaaaaa@58EE@

where *H*(*A*) is the source uncertainty of language *A*.

Suppose the system to be set in some reference configuration *A*_0_.

To obtain the unperturbed dynamics of that state, impose a Legendre transform using this derivative, defining another scalar

*S *≡ *H *- ℳ
 MathType@MTEF@5@5@+=feaafiart1ev1aaatCvAUfKttLearuWrP9MDH5MBPbIqV92AaeXatLxBI9gBamrtHrhAL1wy0L2yHvtyaeHbnfgDOvwBHrxAJfwnaebbnrfifHhDYfgasaacH8akY=wiFfYdH8Gipec8Eeeu0xXdbba9frFj0=OqFfea0dXdd9vqai=hGuQ8kuc9pgc9s8qqaq=dirpe0xb9q8qiLsFr0=vr0=vr0dc8meaabaqaciaacaGaaeqabaWaaeGaeaaakeaaimaacqWFZestaaa@3790@*dH*/*d*ℳ
 MathType@MTEF@5@5@+=feaafiart1ev1aaatCvAUfKttLearuWrP9MDH5MBPbIqV92AaeXatLxBI9gBamrtHrhAL1wy0L2yHvtyaeHbnfgDOvwBHrxAJfwnaebbnrfifHhDYfgasaacH8akY=wiFfYdH8Gipec8Eeeu0xXdbba9frFj0=OqFfea0dXdd9vqai=hGuQ8kuc9pgc9s8qqaq=dirpe0xb9q8qiLsFr0=vr0=vr0dc8meaabaqaciaacaGaaeqabaWaaeGaeaaakeaaimaacqWFZestaaa@3790@.     (10)

The simplest possible Onsager relation – again an empirical equation like a regression model – in this case becomes

*d*ℳ
 MathType@MTEF@5@5@+=feaafiart1ev1aaatCvAUfKttLearuWrP9MDH5MBPbIqV92AaeXatLxBI9gBamrtHrhAL1wy0L2yHvtyaeHbnfgDOvwBHrxAJfwnaebbnrfifHhDYfgasaacH8akY=wiFfYdH8Gipec8Eeeu0xXdbba9frFj0=OqFfea0dXdd9vqai=hGuQ8kuc9pgc9s8qqaq=dirpe0xb9q8qiLsFr0=vr0=vr0dc8meaabaqaciaacaGaaeqabaWaaeGaeaaakeaaimaacqWFZestaaa@3790@/*dt *= *LdS*/*d*ℳ
 MathType@MTEF@5@5@+=feaafiart1ev1aaatCvAUfKttLearuWrP9MDH5MBPbIqV92AaeXatLxBI9gBamrtHrhAL1wy0L2yHvtyaeHbnfgDOvwBHrxAJfwnaebbnrfifHhDYfgasaacH8akY=wiFfYdH8Gipec8Eeeu0xXdbba9frFj0=OqFfea0dXdd9vqai=hGuQ8kuc9pgc9s8qqaq=dirpe0xb9q8qiLsFr0=vr0=vr0dc8meaabaqaciaacaGaaeqabaWaaeGaeaaakeaaimaacqWFZestaaa@3790@,     (11)

where *t *is the time and *dS*/*d*ℳ
 MathType@MTEF@5@5@+=feaafiart1ev1aaatCvAUfKttLearuWrP9MDH5MBPbIqV92AaeXatLxBI9gBamrtHrhAL1wy0L2yHvtyaeHbnfgDOvwBHrxAJfwnaebbnrfifHhDYfgasaacH8akY=wiFfYdH8Gipec8Eeeu0xXdbba9frFj0=OqFfea0dXdd9vqai=hGuQ8kuc9pgc9s8qqaq=dirpe0xb9q8qiLsFr0=vr0=vr0dc8meaabaqaciaacaGaaeqabaWaaeGaeaaakeaaimaacqWFZestaaa@3790@. represents an analog to the thermodynamic force in a chemical system. This is seen as acting on the reference state *A*_0_. For

dS/dℳ|A0=0,d2S/dℳ2|A0>0     (12)
 MathType@MTEF@5@5@+=feaafiart1ev1aaatCvAUfKttLearuWrP9MDH5MBPbIqV92AaeXatLxBI9gBaebbnrfifHhDYfgasaacH8akY=wiFfYdH8Gipec8Eeeu0xXdbba9frFj0=OqFfea0dXdd9vqai=hGuQ8kuc9pgc9s8qqaq=dirpe0xb9q8qiLsFr0=vr0=vr0dc8meaabaqaciaacaGaaeqabaqabeGadaaakeaafaqabeGabaaabaGaemizaqMaem4uamLaei4la8Iaemizaq2enfgDOvwBHrxAJfwnHbqeg0uy0HwzTfgDPnwy1aaceaGae83mH0KaeiiFaW3aaSbaaSqaaiabdgeabnaaBaaameaacqaIWaamaeqaaaWcbeaakiabg2da9iabicdaWiabcYcaSaqaaiabdsgaKnaaCaaaleqabaGaeGOmaidaaOGaem4uamLaei4la8IaemizaqMae83mH00aaWbaaSqabeaacqaIYaGmaaGccqGG8baFdaWgaaWcbaGaemyqae0aaSbaaWqaaiabicdaWaqabaaaleqaaOGaeyOpa4JaeGimaadaaiaaxMaacaWLjaWaaeWaaeaacqaIXaqmcqaIYaGmaiaawIcacaGLPaaaaaa@55C9@

the system is quasistable, a Black hole, if you will, and externally imposed forcing mechanisms will be needed to effect a transition to a different state. We shall explore this circumstance below in terms of the concept of ecosystem resilience.

Conversely, changing the direction of the second condition, so that

dS2/dℳ2|A0<0,
 MathType@MTEF@5@5@+=feaafiart1ev1aaatCvAUfKttLearuWrP9MDH5MBPbIqV92AaeXatLxBI9gBaebbnrfifHhDYfgasaacH8akY=wiFfYdH8Gipec8Eeeu0xXdbba9frFj0=OqFfea0dXdd9vqai=hGuQ8kuc9pgc9s8qqaq=dirpe0xb9q8qiLsFr0=vr0=vr0dc8meaabaqaciaacaGaaeqabaqabeGadaaakeaacqWGKbazcqWGtbWudaahaaWcbeqaaiabikdaYaaakiabc+caViabdsgaKnrtHrhAL1wy0L2yHvtyaeHbnfgDOvwBHrxAJfwnaGabaiab=ntinnaaCaaaleqabaGaeGOmaidaaOGaeiiFaW3aaSbaaSqaaiabdgeabnaaBaaameaacqaIWaamaeqaaaWcbeaakiabgYda8iabicdaWiabcYcaSaaa@4551@

leads to a repulsive peak, a White hole, representing a possibly unattainable realm of states.

Explicit parametization of ℳ
 MathType@MTEF@5@5@+=feaafiart1ev1aaatCvAUfKttLearuWrP9MDH5MBPbIqV92AaeXatLxBI9gBamrtHrhAL1wy0L2yHvtyaeHbnfgDOvwBHrxAJfwnaebbnrfifHhDYfgasaacH8akY=wiFfYdH8Gipec8Eeeu0xXdbba9frFj0=OqFfea0dXdd9vqai=hGuQ8kuc9pgc9s8qqaq=dirpe0xb9q8qiLsFr0=vr0=vr0dc8meaabaqaciaacaGaaeqabaWaaeGaeaaakeaaimaacqWFZestaaa@3790@ introduces standard – and quite considerable – notational complications (e.g. [56,57]): Imposing a metric for different cognitive dual languages parametized by **K **leads to Riemannian, or even Finsler, geometries, including the usual geodesies (e.g. [55]).

We have defined a new groupoid for the system based on a particular set of equivalence classes of information sources dual to cognitive processes. That groupoid parsimoniously characterizes the available dynamical manifolds, and, in precisely the sense of the earlier development, breaking of the groupoid symmetry creates more complex objects of considerable interest, which will be studied below. This leads to the possibility, indeed, the necessity, of *Deus ex Machina *executive mechanisms forcing transitions between the different possible modes within and across dynamic manifolds.

Equivalence classes of states gave dual information sources. Equivalence classes of information sources give different characteristic system dynamics. Later we will examine equivalence classes of paths within dynamic manifolds, which will produce different directed homotopy topologies characterizing them. This introduces the possibility of different quasi-stable resilience modes *within *individual dynamic manifolds. Ultimately we are identifying a topology which permits the patching-together of substructures into larger composites.

The next important structural iteration, however, is, in some respects, significantly more complicated than a differentiable manifold.

### 9. The rate distortion manifold

The second order iteration above – analogous to expanding the General Linear Model to the Hierarchical Linear Model – which involved paths in parameter space, can itself be significantly extended. This produces a generalized tunable retina model which can be interpreted as a Rate Distortion Manifold, a concept which further opens the way for import of tools from geometry and topology.

Suppose, now, that threshold behavior for institutional reaction requires some elaborate system of nonlinear relationships defining a set of renormalization parameters Ω_*k *_≡ ω1k
 MathType@MTEF@5@5@+=feaafiart1ev1aaatCvAUfKttLearuWrP9MDH5MBPbIqV92AaeXatLxBI9gBaebbnrfifHhDYfgasaacH8akY=wiFfYdH8Gipec8Eeeu0xXdbba9frFj0=OqFfea0dXdd9vqai=hGuQ8kuc9pgc9s8qqaq=dirpe0xb9q8qiLsFr0=vr0=vr0dc8meaabaqaciaacaGaaeqabaqabeGadaaakeaaiiGacqWFjpWDdaqhaaWcbaGaeGymaedabaGaem4AaSgaaaaa@30FC@, ..., ωmk
 MathType@MTEF@5@5@+=feaafiart1ev1aaatCvAUfKttLearuWrP9MDH5MBPbIqV92AaeXatLxBI9gBaebbnrfifHhDYfgasaacH8akY=wiFfYdH8Gipec8Eeeu0xXdbba9frFj0=OqFfea0dXdd9vqai=hGuQ8kuc9pgc9s8qqaq=dirpe0xb9q8qiLsFr0=vr0=vr0dc8meaabaqaciaacaGaaeqabaqabeGadaaakeaaiiGacqWFjpWDdaqhaaWcbaGaemyBa0gabaGaem4AaSgaaaaa@316F@. The critical assumption is that there is a tunable zero order state, and that changes about that state are, in first order, relatively small, although their effects on punctuated process may not be at all small. Thus, given an initial *m*-dimensional vector Ω_*k*_, the parameter vector at time *k *+ 1, Ω_*k*+1_, can, in first order, be written as

Ω_*k*+1 _≈ **R**_*k*+1_Ω_*k*_,     (13)

where **R**_*t*+1 _is an *m *× *m *matrix, having *m*^2 ^components.

If the initial parameter vector at time *k *= 0 is Ω_0_, then at time *k*

Ω_*k *_= **R**_*k*_**R**_*k*-1_...**R**_1_Ω_0_.     (14)

The interesting correlates of individual or collective consciousness are, in this development, *now represented by an information-theoretic path defined by the sequence of operators ***R**_*k*_, each member having *m*^2 ^components. The grammar and syntax of the path defined by these operators is associated with a dual information source, in the usual manner.

The effect of an information source of external signals, **Y**, is now seen in terms of more complex joint paths in *Y *and *R*-space whose behavior is, again, governed by a mutual information splitting criterion according to the JAEPT.

The complex sequence in *m*^2^-dimensional *R*-space has, by this construction, been projected down onto a parallel path, the smaller set of *m*-dimensional *ω*-parameter vectors Ω_0_, ..., Ω_*k*_.

If the punctuated tuning of institutional attention is now characterized by a 'higher' dual information source – an embedding generalized language -so that the paths of the operators **R**_*k *_are autocorrelated, then the autocorrelated paths in Ω_*k *_represent output of a parallel information source which is, given Rate Distortion limitations, apparently a grossly simplified, and hence highly distorted, picture of the process represented by the *R*-operators, having *m *as opposed to *m *× *m *components.

High levels of distortion may not necessarily be the case for such a structure, *provided it is properly tuned to the incoming signal*. If it is inappropriately tuned, however, then distortion may be extraordinary.

The next step is to examine a single iteration in detail, assuming now there is a (tunable) zero reference state, **R**_0_, for the sequence of operators **R**_*k*_, and that

Ω_*k*+1 _= (**R**_0 _+ *δ***R**_*k*+1_)Ω_*k*_,         (15)

where *δ***R**_*k *_is small in some sense compared to **R**_0_.

Note that in this analysis the operators **R**_*k *_are, implicitly, determined by linear regression. It is thus possible to invoke a quasi-diagonalization in terms of **R**_0_. Let **Q **be the matrix of eigenvectors which Jordan-block-diagonalizes **R**_0_. Then

**Q**Ω_*k*+1 _= (**QR**_0_**Q**^-1 ^+ **Q***δ***R**_*k*+1_**Q**^-1^)**Q**Ω_*k*_.     (16)

If **Q**Ω_*k *_is an eigenvector of **R**_0_, say *Y*_*j *_with eigenvalue *λ*_*j*_, it is possible to rewrite this equation as a generalized spectral expansion

Yk+1=(J+δJk+1)Yj≡λjYj+δYk+1=λjYj+∑i=1naiYi.     (17)
 MathType@MTEF@5@5@+=feaafiart1ev1aaatCvAUfKttLearuWrP9MDH5MBPbIqV92AaeXatLxBI9gBaebbnrfifHhDYfgasaacH8akY=wiFfYdH8Gipec8Eeeu0xXdbba9frFj0=OqFfea0dXdd9vqai=hGuQ8kuc9pgc9s8qqaq=dirpe0xb9q8qiLsFr0=vr0=vr0dc8meaabaqaciaacaGaaeqabaqabeGadaaakeaafaqabeGabaaabaGaemywaK1aaSbaaSqaaiabdUgaRjabgUcaRiabigdaXaqabaGccqGH9aqpdaqadaqaaGqabiab=PeakjabgUcaRGGaciab+r7aKjab=PeaknaaBaaaleaacqWGRbWAcqGHRaWkcqaIXaqmaeqaaaGccaGLOaGaayzkaaGaemywaK1aaSbaaSqaaiabdQgaQbqabaGccqGHHjIUcqGF7oaBdaWgaaWcbaGaemOAaOgabeaakiabdMfaznaaBaaaleaacqWGQbGAaeqaaOGaey4kaSIae4hTdqMaemywaK1aaSbaaSqaaiabdUgaRjabgUcaRiabigdaXaqabaaakeaacqGH9aqpcqGF7oaBdaWgaaWcbaGaemOAaOgabeaakiabdMfaznaaBaaaleaacqWGQbGAaeqaaOGaey4kaSYaaabCaeaacqWGHbqydaWgaaWcbaGaemyAaKgabeaakiabdMfaznaaBaaaleaacqWGPbqAaeqaaaqaaiabdMgaPjabg2da9iabigdaXaqaaiabd6gaUbqdcqGHris5aOGaeiOla4caaiaaxMaacaWLjaWaaeWaaeaacqaIXaqmcqaI3aWnaiaawIcacaGLPaaaaaa@680C@

**J **is a block-diagonal matrix, *δ***J**_*k*+1 _≡ **QR**_*k*+1_**Q**^-1^, and *δY*_*k*+1 _*has been expanded in terms of a spectrum of the eigenvectors of ***R**_0_, with

|*a*_*i*_| ≪ |*λ*_*j*_|, |*a*_*i*+1_| ≪ |*a*_*i*_|.     (18)

The point is that, provided **R**_0 _has been tuned so that this condition is true, the first few terms in the spectrum of this iteration of the eigenstate will contain most of the essential information about *δ***R**_*k*+1_. This appears quite similar to the detection of color in the retina, where three overlapping non-orthogonal eigenmodes of response are sufficient to characterize a huge plethora of color sensation. Here, if such a tuned spectral expansion is possible, a very small number of observed eigenmodes would suffice to permit identification of a vast range of changes, so that the rate-distortion constraints become quite modest. That is, there will not be much distortion in the reduction from paths in *R*-space to paths in Ω-space. Inappropriate tuning, however, can produce very marked distortion, even institutional inattentional blindness, in spite of multitasking.

Wallace [14] describes the individual case as follows:

"Conscious attention acts through a Rate Distortion manifold, a kind of retina-like filter for grammatical and syntactical meaningful paths, which affects what can be brought to consciousness in a punctuated manner akin to a phase transition. Signals outside the topologically constrained tunable syntax/grammar bandpass of this manifold are subject to lessened probability of punctuated conscious detection: generalized [inattentional blindness]."

Note that higher order Rate Distortion Manifolds are likely to give better approximations than lower ones, in the same sense that second order tangent structures give better, if more complicated, approximations in conventional differentiable manifolds (e.g. [58]). Nonetheless, inattentional blindness remains a canonical failure mode for all individually or collectively conscious systems, although it may be lessened in the latter case if individual workspaces are sufficiently numerous and diverse – multiple, significantly different **R**_0_'s – and are able to cross-communicate with little distortion.

Indeed, Rate Distortion Manifolds can be quite formally described using standard techniques from topological manifold theory [15]. The essential point is that a rate distortion manifold is a topological structure which constrains the 'stream of institutional collective consciousness' as well as the pattern of communication between institutional giant components, much the way a riverbank constrains the flow of the river it contains. This is a fundamental insight, which we pursue further.

### 10. Institutional resilience

The groupoid treatment of modular cognitive networks above defined equivalence classes of *states *according to whether they could be linked to some origin by grammatical/syntactical high probability meaningful paths, and equivalence classes of *languages *according to their empirically-characterized dynamical properties. One can ask the precisely complementary question regarding *paths *within a given dynamic manifold: For any two particular given states, is there some sense in which it is possible to define equivalence classes across the set of meaningful paths linking them? This will give rise to the fundamental topological groupoid of a particular cognitive dynamic manifold.

This is of particular interest to the second order hierarchical model which, in effect, describes a universality class tuning of the renormalization parameters characterizing the dancing, flowing, tunably punctuated accession to individual or collective consciousness.

A closely similar question is central to recent algebraic geometry approaches to concurrent, i.e. highly parallel, computing (e.g. [59-61]), which we adapt.

**Figure 3:**
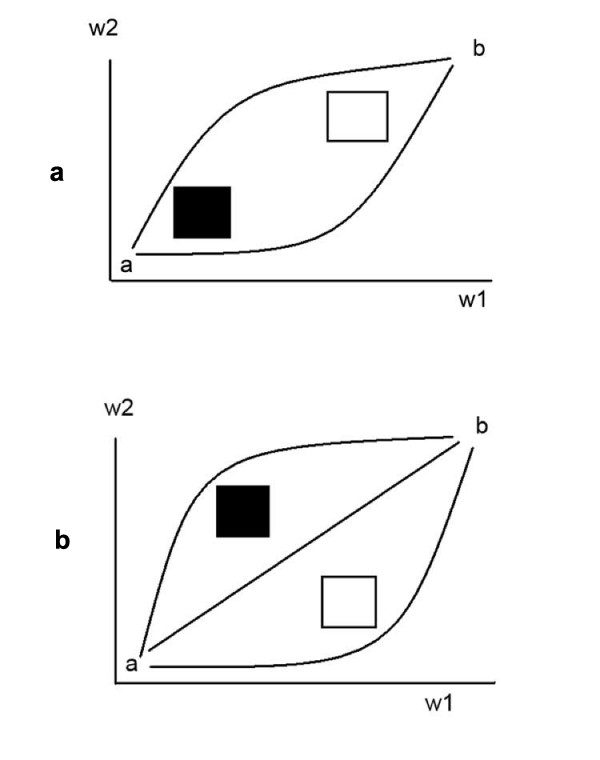
**a**. Diagonal Black and White holes in the two dimensional *ω*-plane. Only two direct paths can link points *a *and *b *which are continuously deformable into one another without crossing either hole. There are two additional monotonic switchback paths which are not drawn. Equivalence classes of paths define the fundamental dihomotopy groupoid. **b**. Cross-diagonal Black and White holes as in 3a. Three direct equivalence classes of continuously deformable paths can link *a *and *b*. Thus the two spaces are topologically distinct, having different dihomotopy groupoids. Here monotonic switchbacks are not possible, although relaxation of that condition can lead to 'backwards' switchbacks and intermediate loopings.

For the moment restrict attention to a giant component system characterized by two renormalization parameters, say *ω*_1 _and *ω*_2 _and consider the set of meaningful paths connecting two particular points, say *a *and *b*, in the two dimensional *ω*-space plane of figure 3. The arguments surrounding equations (6), (7) and (12) suggests that there may be regions of fatal attraction and strong repulsion, Black holes and White holes, which can either trap or deflect the path of institutional cognition.

Figures 3a and 3b show two possible configurations for a Black and a White hole, diagonal and cross-diagonal. If one requires path monotonicity – always increasing or remaining the same – then, following, [61], figs. 6, 7, there are, intuitively, two direct ways, without switchbacks, that one can get from *a *to *b *in the diagonal geometry of figure 3a, without crossing a Black or White hole, but there are three in the cross-diagonal structure of figure 3b.

Elements of each 'way' can be transformed into each other by continuous deformation without crossing either the Black or White hole. Figure 3a has two additional possible monotonic ways, involving over/under switchbacks, which are not drawn. Relaxing the monotonicity requirement generates a plethora of other possibilities, e.g. loopings and backwards switchbacks, but it is not clear under what circumstances such complex paths can be meaningful.

These ways are the equivalence classes generating the fundamental topological groupoid of the two different *ω*-spaces, analogs to the fundamental homotopy groups in spaces which admit of loops (e.g. [62]). The closed loops needed for classical homotopy theory are impossible for this kind of system because of the 'flow of time' defining the output of an information source -one goes from *a *to *b*, although, for nonmonotonic paths, intermediate looping would seem possible. The theory is thus one of directed homotopy, dihomotopy, and the central question revolves around the continuous deformation of paths in *ω*-space into one another, without crossing Black or White holes. Goubault and Rausssen [60] provide another introduction to the formalism.

These ideas can, of course, be applied to lower level cognitive modules as well as to the second order hierarchical cognitive model of institutional cognition where they are, perhaps, of more central interest.

Empirical study will likely show how the influence of cultural heritage or developmental history defines quite different dihomotopies of attentional focus in human organizations. That is, the topology of blind spots and their associated patterns of perceptual completion in human organizations will be culturally or developmentally modulated. It is this developmental cultural topology of multitasking organization attention which, acting in concert with the inherent limitations of the rate distortion manifold, generates the pattern of organizational inattentional blindness.

Such considerations, and indeed the Black Hole development of equation (12), suggest that a multitasking organization which becomes trapped in a particular pattern of behavior cannot, in general, expect to emerge from it in the absence of some forcing mechanism.

This form of behavior is central to ecosystem resilience theory, which we will examine at two different scales. The first is at the topology of an individual dynamic manifold. The second emerges when the dynamical groupoid is broken by hierarchical linkages which patch together different manifolds. These may, in fact, simply represent different scales of the same basic phenomenon, i.e. a patching of spaces.

Ecosystem theorists, in fact, recognize several different kinds of resilience (e.g. [63]). The first, which they call 'engineering resilience', since it is particularly characteristic of machines and man-machine interactions, involves the rate at which a disturbed system returns to a presumed single, stable, equilibrium condition, following perturbation. From that limited perspective, a resilient system is one which quickly returns to its one stable state.

Not many biological or social phenomena seem resilient in this simplistic sense.

Holling's [64] particular contribution was to recognize that sudden transitions between different, at best quasi-stable, domains of relation among ecosystem variates were possible, i.e. that more than one 'stable' state was possible for real ecosystems. Gunderson [63] puts the matter as follows:

"One key distinction between these two types of resilience lies in assumptions regarding the existence of multiple [quasi-] stable states. If it is assumed that only one stable state exists or can be designed to exist, then the only possible definition and measures for resilience are near equilibrium ones – such as characteristic return time... The concept of ecological resilience presumes the existence of multiple stability domains and the tolerance of the system to perturbations that facilitate transitions among stable states. Hence, ecological resilience refers to the width or limit of a stability domain and is defined by the magnitude of disturbance that a system can absorb before it changes stable states... The presence of multiple [quasi-] stable states and transitions among them [has] been [empirically] described in a [large] range of ecological systems..."

The topology of institutional cognition provides a tool for study of resilience in human organizations or social systems. The obvious conjecture is that the set of equivalence classes of directed homotopy described above formally classifies quasi-equilibrium states, and thus characterizes the different possible ecosystem resilience modes by their fundamental topological groupoids within a particular dynamic manifold. However, a shift between dynamical manifolds would represent a qualitatively different kind of resilience transition.

This approach generalizes some current work on distributed cognition (e.g. [65]) in that it is not restricted to engineering resilience, i.e. graceful degradation, followed by return to equilibrium, but encompasses the idea of pathological states much like the eutrophication of a pristine lake. Changes between orders of these quasi-equilibrium states, in our model, require more than simply the lessening of challenge, but positive, intensive, intervention from outside the system itself to shift domains of quasi-stability.

Ultimately, we are invoking the necessity of 'executive force' to move organizations between different modes, either within one, or between, dynamic manifolds. If there is a insufficient repertory of possibilities, or insufficient ability to cause transition, then 'market forces' may be literally devastating, as was the experience of the Columbia space shuttle disaster [66].

Later we will be concerned with the impact of widespread collective stress – disaster – on institutional resilience, but some further analytic machinery is needed.

### 11. Irreversible variation and selection

Collective consciousness can, then, occur across a great variety of institutions, which themselves are culturally constrained, and will persist even as underlying structures may shift. Within an evolutionary setting this would be equivalent to polyphyletic parallelism, where many different responses to similar selection pressures produce similar functional outcomes – for example bird, bat, and insect wings all used for flight. Institutional economics, in fact, takes an explicitly evolutionary view of these matters, (e.g. [67]), abandoning an equilibrium perspective for developmental irreversibility.

Selection pressures write distorted images of themselves onto genetic structure through natural selection [53]. Wallace and Wallace [68,69], in fact, argued that, due to its highly structured nature, an embedding environment constitutes an information source which, as it becomes more closely linked to an organism – as the organism's homeostatic elasticity fails – writes its distorted genetic image as a phase transition, accounting directly for punctuated equilibrium in the fossil record. Analogs with ecological or institutional resilience seem clear.

The essence of evolutionary process, then, is the punctuated occurrence of major innovations in structure and function, which then develop according to an irreversible bush-like branching, that is then pruned by some combination of selection pressure and blind chance. One example is the many mainframe computer companies which flowered and failed, leaving IBM as the principal legacy. Mainframes now face extinction. Another is the many personal computer operating systems that collapsed, leaving Microsoft's version, which itself now faces strong selection pressure and possible extinction.

Thus the various forms of collectively  conscious institutions all encounter 'market' selection pressures and the vicissitudes of chance, and engage in variation through learning and random change, providing another example of irreversible evolutionary process. Selection pressures – market demands -will write images of themselves onto collectively conscious institutions, which must then homeostatically adjust, structurally adapt, or fail. D. Wallace and R. Wallace [70], for example, provide an explicit evolutionary perspective on how the 'South Bronx' process of policy-driven contagious urban decay constituted a draconian selection pressure for social network structure, the basic skeleton upon which any local collectively conscious institution must be built.

Collectively conscious institutions, then, are subject to irreversible evolutionary development, constrained by the intertwining of cultural and historical context which limit adaptability and may well predispose them to characteristic failure modes, which we now explore in more detail.

## Pathologies of individual consciousness

Some insight regarding failure modes in collectively conscious institutions can be gained from the study of pathologies in a system having but a single broadcast workspace, i.e. the human mind [12]. The result is not at all reassuring.

Mental disorders in humans are not well understood. Indeed, such classifications as the *Diagnostic and Statistical Manual of Mental Disorders – fourth edition*, [71], the standard descriptive nosology in the US, have been characterized as prescientific by Gilbert [72] and others. Arguments from genetic determinism fail, in part because of an apparently draconian population bottleneck which, early in our species' history, resulted in an overall genetic diversity less than that observed within and between contemporary chimpanzee subgroups. Arguments from psychosocial stress fare better, but are affected by the apparently complex and contingent developmental paths determining the onset of schizophrenia – one of the most prevalent serious mental disorders – dementias, psychoses, and so forth, some of which may be triggered in utero by exposure to infection, low birthweight, or other stressors.

Gilbert suggests an extended evolutionary perspective, in which evolved mechanisms like the 'flight-or-fight' response are inappropriately excited or suppressed, resulting in such conditions as anxiety or post traumatic stress disorders. Nesse [73] suggests that depression may represent the dysfunction of an evolutionary adaptation which down-regulates foraging activity in the face of unattainable goals.

Kleinman and Good, however, ([74], p. 492) have outlined some of the cross cultural subtleties affecting the study of depression which seem to argue against any simple evolutionary interpretation:

"When culture is treated as a constant (as is common when studies are conducted in our own society), it is relatively easy to view depression as a biological disorder, triggered by social stressors in the presence of ineffective support, and reflected in a set of symptoms or complaints that map back onto the biological substrate of the disorder... However, when culture is treated as a significant variable, for example, when the researcher seriously confronts the world of meaning and experience of members of non-Western societies, many of our assumptions about the nature of emotions and illness are cast in sharp relief. Dramatic differences are found across cultures in the social organization, personal experience, and consequences of such emotions as sadness, grief, and anger, of behaviors such as withdrawal or aggression, and of psychological characteristics such as passivity and helplessness or the resort to altered states of consciousness. They are organized differently as psychological realities, communicated in a wide range of idioms, related to quite varied local contexts of power relations, and are interpreted, evaluated, and responded to as fundamentally different meaningful realities... Depressive illness and dysphoria are thus not only interpreted differently in non-Western societies and across cultures; they are *constituted *as fundamentally different forms of social reality."

More generally, Kleinman and Cohen [75] find that

"[S]everal myths... have become central to psychiatry... The first is that the forms of mental illness everywhere display similar degrees of prevalence... [Second is] an excessive adherence to a principle known as the pathogenic/pathoplastic dichotomy, which holds that biology is responsible for the underlying structure of a malaise, whereas cultural beliefs shape the specific ways in which a person experiences it. The third myth maintains that various unusual culture-specific disorders whose biological bases are uncertain occur only in exotic places outside the West... In an effort to base psychiatry in 'hard' science and thus raise its status to that of other medical disciplines, psychiatrists have narrowly focused on the biological underpinnings of mental disorders while discounting the importance of such 'soft' variables as culture and socioeconomic status..."

Further, serious mental disorders in humans are often comorbid among themselves – depression and anxiety, compulsive behaviors, psychotic ideation, etc. – and with serious chronic physical conditions such as coronary heart disease, atherosclerosis, diabetes, hypertension, dyslipidemia, and so on. These too are increasingly recognized as developmental in nature (e.g. [11]), and are frequently compounded by behavioral problems like violence or substance use and abuse. Indeed, smoking, alcohol and drug addiction, compulsive eating, and the like, are often done as self-medication for the impacts of psychosocial and other stressors, constituting socially-induced 'risk behaviors' which synergistically accelerate a broad spectrum of mental and physical problems.

Recent research on schizophrenia, dyslexia, and autism, supports a 'brain connectivity' model for these disorders which is of considerable interest from a global workspace perspective, since large-scale brain connectivity is essential for the operation of consciousness, a principal, and very old, evolutionary adaptation in higher animals.

Burns et al. [76], on the basis of sophisticated diffusion tensor magnetic resonance imaging studies, find that schizophrenia is a disorder of large-scale neurocognitive networks rather than specific regions, and that pathological changes in the disorder should be sought at the supra-regional level. Both structural and functional abnormalities in frontoparietal networks have been described and may constitute a basis for the wide range of cognitive functions impaired in the disorder, such as selective attention, language processing and attribution of agency.

Silani et al. [77] find that, for dyslexia, altered activation observed within the reading system is associated with altered density of grey and white matter of specific brain regions, such as the left middle and inferior temporal gyri and left arcuate fasciculus. This supports the view that dyslexia is associated with both local grey matter dysfunction and with altered [larger scale] connectivity among phonological/reading areas.

Villalobos et al. [78] explore the hypothesis that large-scale abnormalities of the dorsal stream and possibly the mirror neuron system, may be responsible for impairments of joint attention, imitation, and secondarily for language delays in autism. Their empirical study showed that those with autism had significantly reduced connectivity with bilateral inferior frontal area 44, which is compatible with the hypothesis of mirror neuron defects in autism. More generally, their results suggest that dorsal stream connectivity in autism may not be fully functional.

Courchesne and Pierce [79] suggest that, for autism, connectivity within the frontal lobe is excessive, disorganized, and inadequately selective, whereas connectivity between frontal cortex and other systems is poorly synchronized, weakly responsive and information impoverished. Increased local but reduced long-distance cortical-cortical reciprocal activity and coupling would impair the fundamental frontal function of integrating information from widespread and diverse systems and providing complex context-rich feedback, guidance and control to lower-level systems.

Coplan [80] has observed a striking pattern of excessive frontal lobe self-connectivity in certain cases of anxiety disorder, and Coplan et al. [81] find that maternal stress can affect long-term hippocampal neurodevelopment in a primate model.

As stated, brain connectivity is the sine qua non of the Global Workspace model of individual human consciousness, and further analysis suggests that these disorders cannot be fully understood in the absence of a functional theory of consciousness, and in particular, of a detailed understanding of the elaborate regulatory mechanisms which must have evolved over the past half billion years to ensure the stability of that most central and most powerful of adaptations.

Distortion of consciousness is not simply an epiphenomenon of the emotional dysregulation which many see as the 'real' cause of mental disorder. Like the pervasive effects of culture, distortion of consciousness lies at the heart of both the individual experience of mental disorder and the effect of it on the embedding of the individual within both social relationships and cultural or environmental milieu. Distortion of consciousness in mental disorders inhibits both routine social interaction and the ability to meet internalized or expected cultural norms, a potentially destabilizing positive feedback. Distortion of consciousness profoundly affects the ability to learn new, or change old, skills in the face of changing patterns of threat or opportunity, perhaps the most critical purpose of the adaptation itself. Distortion of consciousness, particularly any decoupling from social and cultural context, is usually a threat to long-term individual survival, and those with mental disorders significantly affecting consciousness typically face shortened lifespans.

## Pathologies of collective consciousness

Human communities, as natural as neighborhoods, or as intentional as a multinational corporation or an army, have, according to the perspective of this work, multiple, effectively simultaneous, global broadcast giant components which must not only function individually, but in concert with, while perhaps in competition for resources with, other similar modules.

Granovetter's [41] Strength of Weak Ties argument is that nondisjunctive relationships, those which do not disjointly partition a community, are essential for efficient function. Strong ties are those which do disjointly partition a group, for example religious affiliation, age cohort, ethnicity, national origin, skin color in some cases, language, and so on. For an institutional setting these might be the classification by Division, Department, Work Group, informal office-politics clique, and so on. From the perspective of GWT, Granovetter's weak ties permit both the formation of individual workspaces, and enable those workspaces to communicate effectively.

Understanding failures within and between institutional global workspaces seems predicated on understanding weak tie structure and dynamics, which are themselves embedded in larger social and cultural contexts. Clearly, individual workspace failures will often be subject to monitoring and control by parallel workspaces, limiting the damage, as it were, in a manner impossible for human consciousness. Thus institutions, if of sufficient internal diversity and able to communicate effectively across that diversity, may suffer less inattentional blindness and less consequence from individual workspace failure, by virtue of parallel operations, but these problems will not be eliminated. Powerful subgroups not subject to contextual constraint seem a particular problem.

Within organizations, several workspaces typically examine a problem, choose possible modes of action, and, essentially, negotiate and reach consensus. If there is a sufficient spectrum of workspace foci – multiple, different **R**_0_'s – and if cross communication between them is not too distorted, a 'good' institutional decision usually emerges.

This is not, however, always the case, and the approach of equations (6) and (7) and their generalization can be used to model some of the myriad possibilities, in particular institutional 'lock-in' to bad procedures, where the system seems to disappear down a black hole, or, conversely, avoids the eight hundred pound gorilla in the livingroom, as it were.

**Figure 4:**
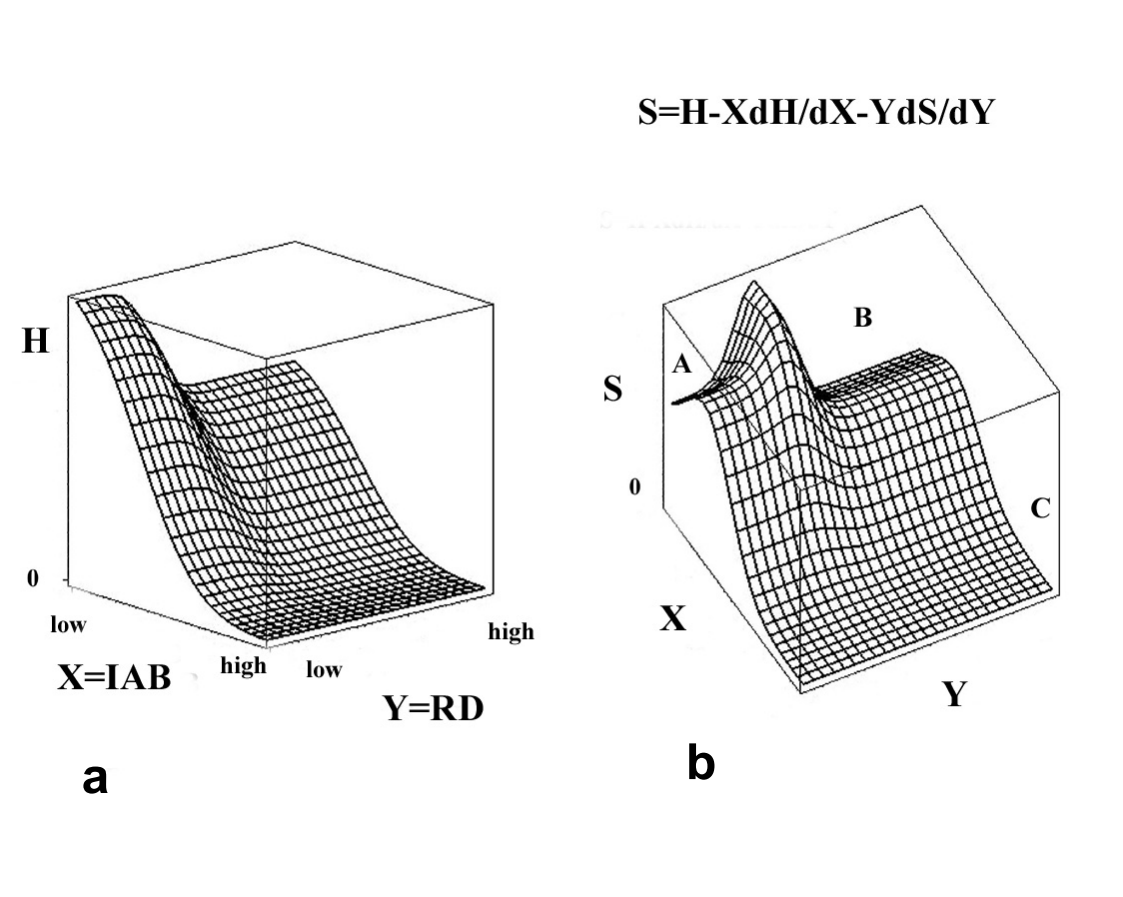
**a**. Source uncertainty, *H*, of the dual information source of institutional collective consciousness, as parametized by degrees of inattentional blindness, *X *= *IAB *and rate distortion *Y *= *RD*. Note the relatively gentle peak at low values of *X*, *Y*. *H *is generated as the product of two error functions. **b**. Generalized Onsager treatment of figure 4a. *S *= *H *(*X*, *Y*) - *XdH*/*dX *- *YdH*/*dY*. The regions marked *A*, *B*, and *C *represent realms of resilient quasi-stablity, divided by barriers defined by the relative peaks in *S*. Transition among them requires a forcing mechanism. From another perspective, limiting resources or imposing social disintegration from the outside – driving down *H *in figure 4a, would drive the system to the lower plain of *C*, in which the system would then become trapped in states having high levels of rate distortion and inattentional blindness.

Suppose we can operationalize and quantify degrees of both institutional inattentional blindness (IAB) and of Rate Distortion (RD) in communication between different institutional global workspaces. This might be done through surveys, structured interviews, statistical characterization of internal telephone patterns, email logs, or the like. The essential assumption is that the dual information source of a collectively conscious institution which has low levels of both IAB and RD will tend to be richer than that of institutions having greater levels. This is shown in figure 4a, where *H *is the source uncertainty, *X *= *IAB*, and *Y *= *RD*. Regions of low *X*, *Y*, i.e. near the origin, have greater source uncertainty than those nearby, so *H *(*X*, *Y*) shows a (relatively gentle) peak at the origin, taken as the product of two error functions. The generalized Onsager argument of equations 6–12 is shown in figure 4b, where *S *= *H *(*X*, *Y*) - *XdH*/*dX *- *YdH*/*dY *is graphed on the *Z *axis against the *X *- *Y *plane, assuming a gentle peak in *H *at the origin. Peaks in *S*, according to the arguments of equations 6–12, constitute repulsive system barriers, which must be overcome by external forces. In figure 4b there are three quasi-stable resilience modes, marked as *A*, *B*, and *C*. The *A *region is locked in to low levels of both inattentional blindness and rate distortion, as it sits in a pocket. Forcing the system in either direction, that is, increasing either IAB or RD, will, initially, be met by homeostatic attempts to return to the resilience state *A*, according to this model.

If, in particular, rate distortion problems become severe in spite of homeostatic mechanisms, the system will then jump to the quasi-stable state *B*, a second pocket. According to the model, however, once that transition takes place, there will be a tendency for the system to remain in a condition of high rate distortion as a matter of institutionalized continued practice. That is, the system will, according to the model, become locked-in to a structure with high distortion in communication between institutional global workspaces, but one having lower overall collective conscious capacity, i.e. a lower value of *H *in figure 4a.

The third pocket, marked *C*, is a broad plain in which both IAB and RD remain high, a highly overfocused, poorly crosslinked, probably pathologically hierarchical, structure which will require significant intervention to alter once it reaches such a quasi-stable resilience mode. Collective conscious capacity, measured by *H *in figure 4a, is the lowest of all for this condition of pathological resilience, and attempts to correct the problem – to return to condition *A*, will be met with very high barriers in *S*, according to figure 4b. That is, mode *C *is very highly resilient, although pathologically so, much like the eutrophication of a pure lake by sewage outflow.

We can argue that the three quasi-equilibrium configurations of figure 4b represent different dynamical manifolds of the system, and that the possibility of transition between them represents the breaking of the associated symmetry groupoid by external forcing mechanisms. That is, three differentiable manifolds representing three different kinds of system dynamics have been patched together by the force of some external executive to create a more complicated topological structure. For cognitive phenomena, this kind of thing is likely to be the rule rather than the exception. 'Pure' groupoids are likely to be arbitrary abstractions, and the fundamental questions will involve the systems of linkages which break the underlying symmetry.

**Figure 5:**
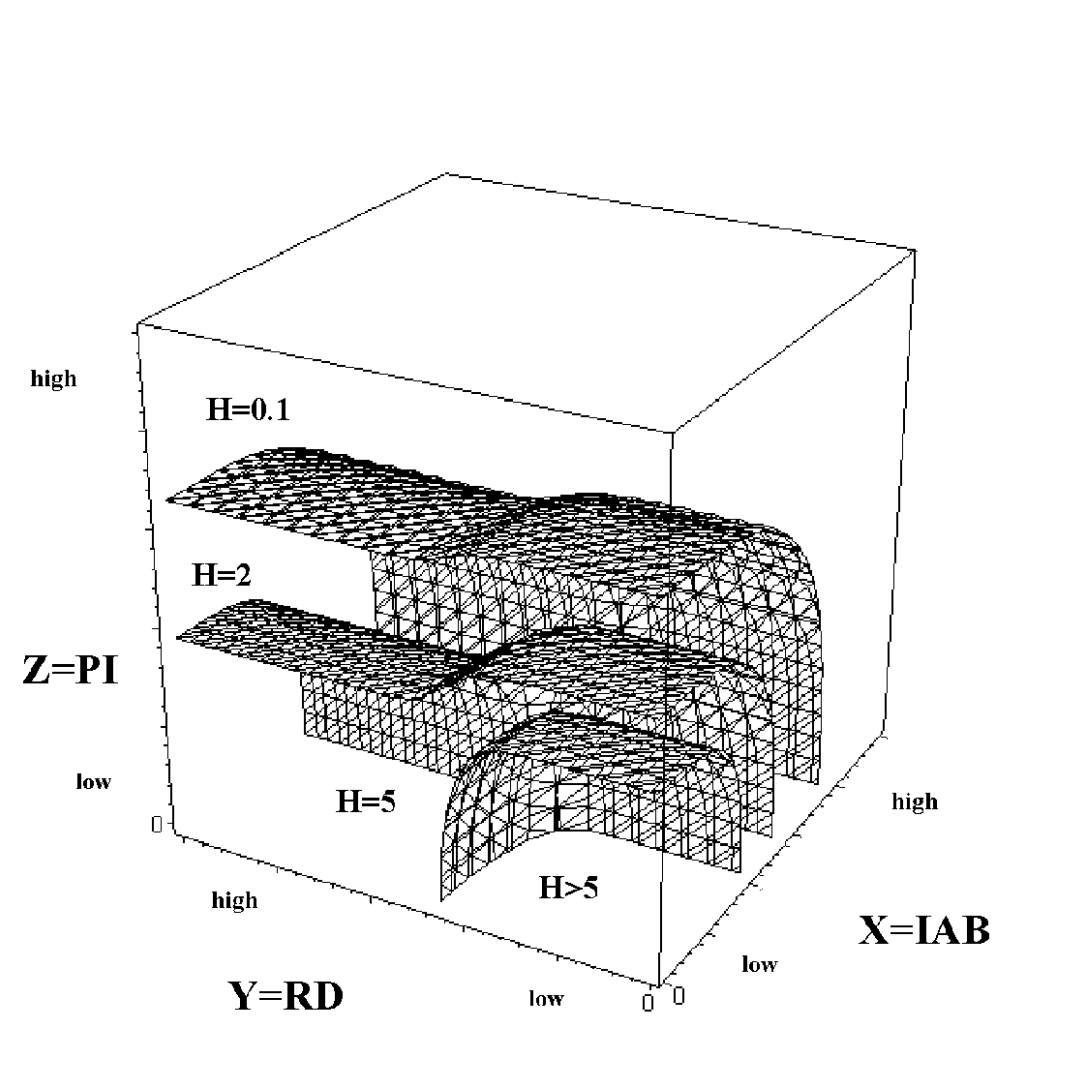
Extension of figure 4a, using the product of three error functions. Assume, now, three characteristic parameters, *X *= *IAB *and *Y *= *RD *as above, with *Z *= *PI *representing the degree of policy/ideological rigidity. Again, the fundamental assumption is that *H *(*X*, *Y*, *Z*) will have a (gentle) peak near the origin. Then *S *= *H *(*X*, *Y*, *Z*) - *XdH*/*dX *- *YdH*/*dY *- *ZdH*/*dZ *has a very complicated system of quasi-stable, locked-in, resilience modes. Again transition between them would require external forcing. Note particularly the nested surfaces with different values of *H*. Attaining low levels of IAB, RD, and PI requires high values of *H*, which, in turn, requires high internal channel capacity, i.e. low social disintegration, and, broadly speaking, high use of energy resources. Institutions suffering social disintegration or resource limitation will be confined to the outer shells, and will experience high levels of pathology. Note that low internal channel capacity – high structural rate distortion within the organization – can, in this model, make it virtually impossible to use even available resources effectively.

Matters are, unfortunately, much more complicated than even this example. Figure 5 shows a three dimensional version of the *X *- *Y *base plane of figures 4a and 4b. Here we assume that the richness of institutional collective consciousness, as measured by its dual information source, is a function of three parameters, *X *= *IAB*, and *Y *= *RD *as before, but now introduce a third parameter, *Z *= *PI*, where *PI *represents the degree to which the institution is driven, not by adaptation to externalities, but by fixed internal policy and/or ideology. The assumption is, again, that institutions less constrained by such factors will be more flexible and have 'richer' cognitive dual information sources. Thus we could again write *H *(*X*, *Y*, *Z*), *S *= *H *- *XdH*/*dX *- *YdH*/*dY *- *ZdH*/*dZ *with *H *'denser' toward the origin of figure 5, i.e. near the of lowest *X*, *Y*, *Z *where *H *has been modeled as the product of three error functions, generalizing figure 4. Quite a large number of quasi-stable resilience modes can result from simple variations of this model, but they cannot, unfortunately, be easily represented in three dimensions.

The variates PI, IAB, and RD have been represented as orthogonal in figures 4 and 5. For real systems, however, related empirical indices are not likely to be independent, so that proper analysis would require application of multivariate statistical methods to produce actual orthogonal measures.

Note that attaining low levels of PI, IAB, and RD in this model requires very high levels of *H*, that is, considerable richness of the dual information source of institutional collective consciousness.

Thus one mechanism likely to impose pathologies of IAB, RD, or PI on a collectively conscious institution would be severe resource limitation. Recall the homology between information source uncertainty and free energy density, equation (5). The dual information source representing a multiple workspace, collectively conscious institution measures, in one broad sense, available 'energy'. Limited resources will impose limits on the possible magnitude of that information source. Constraints on *H *generate structures much like the outer shell of figure 5: *H *< 0.1 generates a system with high rates of the three pathologies. More formally, one might apply Lagrange multiplier or other quantitative constraint/optimization strategies.

A second perspective to limits on *H*, and hence to the imposition of constraints due to RD, IAB, or PI, is derived entirely from communication theory. If one imagines the possibilities for information transmission within the institution to be limited by a maximum channel capacity *C*, which may well be, but is not necessarily, resource-related, then classical information theory [33-35] requires that the dual information source transmitting along the channel must satisfy the relation

*H*(*X*, *Y*, *Z*) ≤ *C*.

Invoking the homology with free energy density, again, heuristically speaking, an institution cannot 'spend energy' at a rate greater than some limiting value *C*, which is the (effective) channel capacity of that institution. Institutions embedded in socially disintegrating subcultures, even if they 'objectively' have resources comparable to similar institutions in more integrated subcultures, will not be able to operate at cognitive rates greater than their channel capacity *C*, which is, effectively, the image of imposed cultural or other constraints.

Thus socially disintegrated subcultures will likely impose capacity limits on embedded collectively conscious institutions which will express themselves as institutional pathologies of rate distortion, inattentional blindness, or rigid policy/ideology, even when given supposedly adequate resources. That is, both embedding social disintegration and limited resources act, effectively, to dumb-down collectively conscious institutions, according to this model.

From an inverse perspective, resource limitation/competition and embedding social disintegration can act synergistically to limit the richness of institutional collective consciousness, thus imposing constraints of rate distortion, inattentional blindness, or policy/ideology on the ability of the organization to recognize patterns of threat or opportunity. That is, imposing limits on maximum *H *in figures 4a or 5 drives the system into the dumbed-down, locked-in realms of *S *– states *B *and *C *and their generalization to higher dimension. Thus, within oppressed communities, IAB, RD, and PI can sometimes be viewed as responses of institutional triage to social disintegration and/or resource competition/limitation. Within oppressor social structures, however, mechanisms of pathological resilience may simply dominate: Apartheid systems can defend themselves.

An important implication of this analysis is that significant collectively experienced adverse events – disasters – which either constrain resources or degrade underlying social communication channels, can trigger shifts in ecosystem resilience domain leading to broad-scale, locked-in, patterns of institutional pathology across an entire community. These may be a change in mode within a single dynamic manifold, or, if the disaster is severe enough, represent a full-scale shift between manifolds. Recovery from disaster-induced institutional pathology, as the next section shows, may be compromised by the very events or forces which caused the disaster.

## Pathologies of therapeutic intervention

Persistent failure by an individual workspace, or a particular subset of them, the effects of inattentional blindness, or pathologies of rate distorted intraworkspace communication, or of policy and ideology, can, in theory, be addressed by external correction, therapeutic intervention by other entities, i.e. the interventionist 'Deus ex Machina'. Pathological context, which is often responsible for global workspace failures of various kinds, can, however, become convoluted with the intervention itself, resulting in therapeutic failure. It is possible to model this in more detail.

Recall that the essential characteristic of cognition in this formalism involves a mapping, *h*(*x*), of a (convolutional) path *x *= *a*_0_, *a*_1_, ..., *a*_*n*_, ... onto a member of one of two disjoint sets, *B*_0 _or *B*_1_. Thus respectively, either (1) *h*(*x*) ∈ *B*_0_, implying no action taken, or (2), *h*(*x*) ∈ *B*_1_, and some particular response is chosen from a large repertoire of possible responses. There is an evident problem in defining these two disjoint sets, suggesting that some higher order, i.e. executive, cognitive module is needed to determine what constitutes *B*_0_, the set of normal actions and procedures, those not constituting explicit intervention. Again, this is because there is no low energy mode for information systems. That is, virtually all states are more or less high energy, high information content or transmission, states. Thus there is no natural way to identify a ground state using the physicist's favorite variational or other minimization arguments.

Suppose that higher order executive cognitive module, which can be described as a kind of Zero Mode Identification, interacts with an embedding, highly structured quasi-language of systemic perturbation – market forces and failures, disasters, structured noise, and the like. Instantiating a Rate Distortion image of that embedding stress, the ZMI begins to map one or more members of the set *B*_1 _into the set *B*_0_, or vice versa, when a circumstance requiring action is ignored. Recurrent hits on that aberrant state would be experienced as episodes of institutional pathology, over or under reaction.

Empirical tests of this hypothesis quickly involve real-world regression models of the interrelations among measurable markers of success and failure, leading to the Rate Distortion Manifold arguments above.

Different eigenmodes *Y*_*k *_of the RDM regression model characterized by the zero mode matrix **R**_0 _can be taken to represent the shifting-of-gears between different languages defining the sets *B*_0 _and *B*_1_. That is, different eigenmodes of the RDM would correspond to different required (and possibly mixed) characteristic systemic responses.

If there is a state (or set of states) *Y*_1 _such that **R**_0_*Y*_1 _= *Y*_1_, then the unitary kernel *Y*_1 _corresponds to the condition 'no response required', i.e. the set *B*_0_.

Suppose pathology becomes manifest, i.e.

**R**_0 _→ **R**_0 _+ *δ***R **≡ R^
 MathType@MTEF@5@5@+=feaafiart1ev1aaatCvAUfKttLearuWrP9MDH5MBPbIqV92AaeXatLxBI9gBaebbnrfifHhDYfgasaacH8akY=wiFfYdH8Gipec8Eeeu0xXdbba9frFj0=OqFfea0dXdd9vqai=hGuQ8kuc9pgc9s8qqaq=dirpe0xb9q8qiLsFr0=vr0=vr0dc8meaabaqaciaacaGaaeqabaqabeGadaaakeaaieqacuWFsbGugaqcaaaa@2DEF@_0_,

so that some chronic 'excited state' becomes the new unitary kernel, and

*Y*_1 _→ Y^
 MathType@MTEF@5@5@+=feaafiart1ev1aaatCvAUfKttLearuWrP9MDH5MBPbIqV92AaeXatLxBI9gBaebbnrfifHhDYfgasaacH8akY=wiFfYdH8Gipec8Eeeu0xXdbba9frFj0=OqFfea0dXdd9vqai=hGuQ8kuc9pgc9s8qqaq=dirpe0xb9q8qiLsFr0=vr0=vr0dc8meaabaqaciaacaGaaeqabaqabeGadaaakeaacuWGzbqwgaqcaaaa@2DF7@_1 _≠ *Y*_1_

R^0Y^1=Y^1.
 MathType@MTEF@5@5@+=feaafiart1ev1aaatCvAUfKttLearuWrP9MDH5MBPbIqV92AaeXatLxBI9gBaebbnrfifHhDYfgasaacH8akY=wiFfYdH8Gipec8Eeeu0xXdbba9frFj0=OqFfea0dXdd9vqai=hGuQ8kuc9pgc9s8qqaq=dirpe0xb9q8qiLsFr0=vr0=vr0dc8meaabaqaciaacaGaaeqabaqabeGadaaakeaaieqacuWFsbGugaqcamaaBaaaleaacqaIWaamaeqaaOGafmywaKLbaKaadaWgaaWcbaGaeGymaedabeaakiabg2da9iqbdMfazzaajaWaaSbaaSqaaiabigdaXaqabaGccqGGUaGlaaa@35DF@

Next, assume other, perhaps embedding, corporate global workspaces induce a sequence of therapeutic counterperturbations – deliberate therapeutic interventions – *δ***T**_*k *_according to the pattern

[R^0+δT1]Y^1=Y1,R^1≡R^0+δT1,[R^1+δT2]Y1=Y2     (19)
 MathType@MTEF@5@5@+=feaafiart1ev1aaatCvAUfKttLearuWrP9MDH5MBPbIqV92AaeXatLxBI9gBaebbnrfifHhDYfgasaacH8akY=wiFfYdH8Gipec8Eeeu0xXdbba9frFj0=OqFfea0dXdd9vqai=hGuQ8kuc9pgc9s8qqaq=dirpe0xb9q8qiLsFr0=vr0=vr0dc8meaabaqaciaacaGaaeqabaqabeGadaaakeaafaqabeWabaaabaGaei4waSfcbeGaf8NuaiLbaKaadaWgaaWcbaGaeGimaadabeaakiabgUcaRGGaciab+r7aKjab=rfaunaaBaaaleaacqaIXaqmaeqaaOGaeiyxa0LafmywaKLbaKaadaWgaaWcbaGaeGymaedabeaakiabg2da9iabdMfaznaaCaaaleqabaGaeGymaedaaOGaeiilaWcabaGaf8NuaiLbaKaadaWgaaWcbaGaeGymaedabeaakiabggMi6kqb=jfaszaajaWaaSbaaSqaaiabicdaWaqabaGccqGHRaWkcqGF0oazcqWFubavdaWgaaWcbaGaeGymaedabeaakiabcYcaSaqaaiabcUfaBjqb=jfaszaajaWaaSbaaSqaaiabigdaXaqabaGccqGHRaWkcqGF0oazcqWFubavdaWgaaWcbaGaeGOmaidabeaakiabc2faDjabdMfaznaaCaaaleqabaGaeGymaedaaOGaeyypa0JaemywaK1aaWbaaSqabeaacqaIYaGmaaaaaOGaaCzcaiaaxMaadaqadaqaaiabigdaXiabiMda5aGaayjkaiaawMcaaaaa@5DB2@

and so on, iteratively, so that, in some sense,

*Y*^*j *^→ *Y*_1_.     (20)

That is, the system, as monitored by the RDM, is driven to its original condition.

It may or may not be possible to have R^
 MathType@MTEF@5@5@+=feaafiart1ev1aaatCvAUfKttLearuWrP9MDH5MBPbIqV92AaeXatLxBI9gBaebbnrfifHhDYfgasaacH8akY=wiFfYdH8Gipec8Eeeu0xXdbba9frFj0=OqFfea0dXdd9vqai=hGuQ8kuc9pgc9s8qqaq=dirpe0xb9q8qiLsFr0=vr0=vr0dc8meaabaqaciaacaGaaeqabaqabeGadaaakeaaieqacuWFsbGugaqcaaaa@2DEF@_0 _→ **R**_0_. That is, actual remediation may not be possible, in which case palliation or control is the therapeutic aim.

The essential point is that the pathological state represented by R^
 MathType@MTEF@5@5@+=feaafiart1ev1aaatCvAUfKttLearuWrP9MDH5MBPbIqV92AaeXatLxBI9gBaebbnrfifHhDYfgasaacH8akY=wiFfYdH8Gipec8Eeeu0xXdbba9frFj0=OqFfea0dXdd9vqai=hGuQ8kuc9pgc9s8qqaq=dirpe0xb9q8qiLsFr0=vr0=vr0dc8meaabaqaciaacaGaaeqabaqabeGadaaakeaaieqacuWFsbGugaqcaaaa@2DEF@_0 _and the sequence of therapeutic interventions *δ***T**_*k*_, *k *= 1, 2,... are interactive and reflective, depending on the regression of the set of vectors *Y*^*j *^to the desired state *Y*_1_, much in the same spirit as Jerne's immunological idiotypic hall of mirrors.

The therapeutic problem revolves around minimizing the difference between *Y*^*k *^and *Y*_1 _over the course of treatment. That difference represents the inextricable convolution of treatment failure with adverse reactions to the course of treatment itself, and failure of compliance, often attributed through social construction by 'provider' to 'patient', i.e. failure of the therapeutic alliance.

It should be obvious that the treatment sequence *δ***T**_*k *_itself is a cognitive path of interventions which has, in turn, a dual information source in the sense previously invoked.

Treatment may, then, interact in the usual Rate Distortion manner with the pathogenic patterns of structured perturbation – market pressures, failures, disasters, resource limitations, embedding channel capacity limits, structured 'red' noise, the burdens of history, and the like – which are, themselves, signals from an embedding information source. Thus treatment failure, adverse reactions, and noncompliance will, of necessity, embody a distorted image of embedding structured perturbations which may indeed be responsible for the primary misfunction.

This coupling would most likely occur in a highly punctuated manner, depending in a quantitative way on the degree of interlinking of the threefold system of affected workgroups, therapeutic interaction, and treatment mode, with that perturbation.

Clearly this is only one example of a much larger spectrum of possibilities. Empirical study would seem necessary at this point to prune down the search tree, as it were, making further analysis practical.

One disturbing implication of this analysis is the apparent difficulty of correcting institutional collective consciousness once it becomes overtly pathological. The general experience of greatly shortened lifespan for most individuals suffering developmental pathologies of global workspace connectivity – schizophrenia, dementia, autism, and the like – suggests that the relentless impact of market forces, which are effectively evolutionary selection pressures, guarantees rapid extinction or merger as the most likely outcome for any systematic, large-scale, organizational cognitive lapses. Western market economies are littered with the corpses of extinct enterprises and economic taxa, and the last century has not been kind to states which possessed, or attempted to gain, colonial empires of various forms, nor to the regions and peoples which suffered occupation under colonial regimes [80,81].

In particular, contexts of social disintegration and resource limitation, individually and likely synergistically, severely limit the possibilities of therapeutic intervention to correct pathologies of institutional collective consciousness.

## Discussion and applications

### 1. General remarks

The groupoid defined by an institution's inherent cognitive modular structure can be broken by intrusion of (rapid) crosstalk from within, and by the imposition of (slower) crosstalk from without – market forces and the embedding culture. The former initiates a set of topologically-determined giant component global broadcasts, in a punctuated manner, while the latter deform the underlying topology of the entire system, the directed homotopy limiting what paths can actually be traversed within a given dynamical manifold. Broken symmetry creates richer phenomena in systems characterized by groupoids, just as it does for those characterized by groups.

Dynamic behavior is similarly characterized by a set of manifolds defining another groupoid whose broken symmetry can patch together even more complex topological structures representing quasi-equilibrium resilience conditions, some of which, like the eutrophication of a pristine lake, will be both highly stable and highly pathological. Transitions between elements of this larger entity represent a different kind of resilience than the transition between topological modes characterizing an individual dynamical manifold.

According to theory, some executive agency is needed to either change modes within a single, or cause transition between different, fundamental cognitive dynamic manifolds.

Disasters, in particular, may be agents generating widespread and persistent pathologies of collective consciousness across a community.

Glazebrook [15] has suggested that, lurking in the background of this basic construction, is what Bak et al. [82] call a groupoid atlas, i.e. an extension of topological manifold theory to groupoid mappings. Also lurking is identification and exploration of the natural groupoid convolution algebra which so often marks these structures (e.g. [36,83]).

Multitasking institutional attention acts through a spectrum of Rate Distortion manifolds, retina-like filters for grammatical and syntactical meaningful paths. Signals outside the topologically constrained tunable syntax/grammar bandpass of this spectrum are subject to lessened probability of punctuated conscious detection: organizational inattentional blindness. This would be seriously exacerbated by lack of diversity within the set of global workspaces, and by distortion in the crosstalk between them. Culture and path-dependent developmental history will, according to this model, in conjunction with resource availability and the effects of embedding social disintegration, profoundly affect the phenomenon by imposing additional topological constraints defining the 'surface' along which this second order behavior can (and cannot) glide.

The focus trade-off can be reexpressed in terms of a syntactical/grammatical version of conventional signal theory, that is, as a tuned meaningful path form of the classic balance between sensitivity and selectivity, as particularly constrained by the directed homotopy or pathological resilience imposed by cultural heritage on a basic institutional experience that is itself the outcome of historical process.

Implicit, however, are the constraints imposed by embedding cultural heritage and institutional history, which may hold the system to a develop-mentally, indeed, Lamarckian evolutionarily, determined topology.

Overall, the analysis is analogous to, but more complicated than, Wallace's version of Baars' Global Workspace theory [11-14]. Intuitively, one suspects that the higher the dimension of the second order attentional Rate Distortion Manifold, that is, the greater the multitasking, the broader the effective bandwidth of attentional focus, and the less likely is inattentional blindness if the spectrum of internal workspaces is sufficiently diverse and well enough interconnected. For a conventional differentiable manifold, a second or higher order tangent space would give a better approximation to the local manifold structure than a simple plane [58].

Similarly, the highly parallel, multitasking set of global workspaces which constitute institutional collective consciousness should be less prone to the consequences of individual workspace failure, provided cross communication between them is relatively undistorted.

As discussed, however, this may not be the case, and very complicated patterns of pathological resilience, institutional lock-in, can become established which would require external intervention for correction. Such therapeutic intervention is itself, however, subject to the very externalities which may have, in fact, created the problem in the first place.

Conversely, pathological resilience can be induced by disasters which limit resources or impose social disintegration.

It is possible to introduce the evolutionary selection pressures of market forces into this model, using the approach of [53], so that internal variation and selection generate an irreversible path dependent developmental process.

Inattentional blindness, while constrained by multitasking, is not eliminated by it. This suggests that higher order institutional cognition, the generalization of individual consciousness, is subject to canonical and idiosyncratic patterns of failure analogous to, but perhaps more subtle than, the kind of disorders described in [12,13]. Indeed, while machines designed along these principles – i.e. rapid, multitasking Global Workspace devices -could be spectacularly efficient at many complex tasks, ensuring their stability might be even more difficult than for intelligent, and hence conscious, machines designed as analogs of the human mind. The relatively slow pace of institutional collective consciousness may well provide significant opportunities for self or externally-induced correction, difficult as that may be in reality, which would be even more difficult for machines operating in the range of a few hundred milliseconds.

Further, the necessity of interaction – synchronous or asynchronous -between institutional giant components suggests the possibility of failures governed by the Rate Distortion Theorem. Forcing rapid communication between institutional global workspaces ensures high levels of average distortion. Recent, and very elegant, ethnographic work by Cohen et al. [84] and Laxmisan et al. [85] regarding systematic medical error in emergency rooms focuses particularly on 'handover' problems at shift change, where incoming medical staff are rapidly briefed by outgoing staff. Systematic information overload in such circumstances then becomes routine, and is widely recognized as a potential error source.

Analogs with mental illness in humans suggest that failures within individual workspaces, while limited by interactional context, will remain serious sources of overall institutional failure, particularly when involving powerful, higher authority modules or work groups not subject to peer-level contextual constraint. Therapeutic intervention can itself reflect the very external perturbations causing individual or large-scale workspace failures. Institutions suffering significant workspace pathologies, like similarly afflicted individuals, are likely to face truncated lifespans in the face of market selection pressures, other relentless externalities, and the deteriorating internal dynamics of corporate dementia.

The hierarchical cognitive model appropriate to institutional collective consciousness is considerably more complicated than for individual consciousness, which, perhaps in a tradeoff permitting rapid, accurate, response to environmental stimulation, seems biologically limited to a single shifting, tunable giant component broadcast structure.

Shared culture seems to provide far more than merely a shared language for the establishment of the human organizations which enable our adaptation to, or alteration of, our varied environments. It also may provide the stabilizing mechanisms needed to overcome many of the canonical and idiosyncratic failure modes inherent to such organizations – inattentional blindness, and pathologies within, and in communication between, individual workspaces, as well as the effects of ideological and policy constraints, and the impact of disasters.

### 2. Similar approaches

In addition to the seminal work of Hutchins and colleagues cited above, there are other analogous perspectives in the literature.

One such is Robert Sampson's 'collective efficacy' approach to community function [86,87]. A large and growing body of sociological research, beginning with Granovetter [41], emphasizes the essential role of nondisjunctive 'weak' social ties within a community. Collective efficacy is a recent reworking of the basic idea [87]:

"...[Collective efficacy [means] an emphasis on shared beliefs in a neighborhood's capability for action to achieve an intended effect, coupled with an active sense of engagement on the part of residents. Some density of social networks is essential... [b]ut the key theoretical point is that *networks have to be activated to be ultimately meaningful*. Collective efficacy therefore helps to elevate the 'agentic' aspect of social life over a perspective centered on the accumulation of stocks of [social capital]. This is consistent with a redefinition of social capital in terms of expectations for action within a collectivity... [in sum] *social networks foster the conditions under which collective efficacy may flourish, but they are not sufficient for the exercise of control*."

From the viewpoint of our analysis, Sampson invokes a version of community cognition, the ability of a neighborhood to perceive patterns of threat or opportunity, to compare those perceived patterns with an internal, shared, picture of the world, and to choose one or a few collective actions from a much larger repertory of those possible, and to carry them out. Disjunctive or 'strong' social ties define some of the underlying cognitive modules – collective and individual – within the neighborhood. Weak ties, then, are those which link such modules – individual or collective – across the community. Individuals, defined subgroups, or formal organizations, may have multiple roles within that community, permitting the formation of multiple global workspaces, if the strength of the various weak ties linking them is sufficient. Institutional cognition, in the sense of this work, emerges as a dynamic, collective phenomenon. Cultural constraints and developmental trajectory serve as 'contexts' to both stabilize and direct the resulting cognitive processes, which may still fail through inattentional blindness, resource limitation, or other pathologies including failure of communication between institutional global workspaces.

This is, however, not Sampson's static, cross-sectional, structure, but, rather, is deeply constrained, not just by shared culture, but by the path dependent historic development of the community itself. Our own work (e.g. [68,88-93]) demonstrates that 'planned shrinkage', 'urban renewal', or other disruptions of weak ties akin to ethnic cleansing, can place neighborhoods onto decades-long irreversible developmental, perhaps evolutionary, trajectories of social disintegration which short-circuit effective community cognition. This is, indeed, a fundamental political purpose of such programs.

There are many other treatments of collective consciousness.

Thomas Burns and his collaborators have, at times, focused particularly on the role of social process in understanding consciousness. Burns and Engdalh [94] write

"What is particularly striking about the academic consciousness industry is the absence of sociology...

A collective has the capacity in its collective representations and communications about what it can (and cannot) do, or should do (or should not do). It monitors its activities, its achievements and failures, and... analyzes and discusses itself as a defined and developing collective agent... a collective has potentially a rich basis not only for talking about, discussing, agreeing (or disagreeing) about a variety of objects... but it also has a means to conceptualize and develop alternative types of social relationships, effective forms of leadership, coordination and control, and... new normative orders and institutional arrangements... These potentialities enable systematic, directed problem solving, and the generation of variety and complex strategies. In particular selective environments, these make for major evolutionary advantages...

[However]..[c]ollective representations and reflectivity and directed problem-solving based on them may prevent human groups from experiencing or discovering the un-represented or un-named; unrecognized or poorly defined problems cannot be dealt with... Reflective and problem-solving powers may then be distorted, the generation of alternatives and varieties narrow and largely ineffective, and social innovation and transformation misdirected and possibly self-destructive..."

Burns and Engdahl [94-96] appear to describe institutional cognition and something much like inattentional blindness. Our innovation is to propose that the particular evolutionary advantage of such cognition is its potential ability to entertain several global workspaces simultaneously, and thus raise overall action capacity while reducing, but not eliminating, inattentional blindness, although introducing failures involving miscommunication between institutional global workspaces.

As discussed earlier, a distributed cognition paradigm has emerged to challenge simple 'mental representation' models in primate behavior studies. Barrett and Henzi [97] comment as follows:

"Physiological studies reveal that the blue fin tuna... should not be able to swim as fast as it actually does. Studies by fluid dynamicists, however, show that tuna are able to find naturally occurring currents in the water, and then use their tails to create additional vortices, which they exploit for extra propulsion.... [T]he 'real swimming machine' is not the tuna alone, but the tuna 'in its proper context' – the tuna, plus the water, plus the vortices it creates and exploits. As for tuna, so for primates: the real 'social intelligence machine' is the primate acting in its proper context – its social group. ... [C]ognition is 'situated' and 'distributed'. Cognition is not limited by the 'skin and skull' of the individual... but uses resources and materials in the environment in the same way that tuna use vortices. The dynamic social interactions of primates... can be investigated as cognitive processes in themselves.... A distributed approach... considers all cognitive processes to emerge from the interactions between individuals, and between individuals and their world... Perhaps our greatest opportunistic and prosocial innovation as group-living animals has been to distribute our cognition to an unprecedented level by storing our essential information in other minds, instead merely of our own."

As Johnson [98] puts it,

"...[A]... paradigm shift... has been taking place in the study of human cognition... from models that focus on internal mental representations to ones that see cognition as a more distributed process – i.e. a process that occurs not just within but *between *individuals... [C]ognition is expanded from an individual enterprise to a distributed activity that involves a variety of socio-cultural elements, including the behavior of multiple individuals, their use of objects, and their shared histories."

As a reviewer has pointed out, these and the related models of Hutchins et al. cited above, are, in fact, representational models in which the representation is itself distributed.

Patel [99] uses the distributed cognition model to describe a paradigm shift regarding the role of technology in medicine:

"The notion of distributed cognition suggests that the immediate physical and social resources outside the person participate in cognition, not just as a source of input and a receiver of output, but as a vehicle of thought... the individual and the environment should be viewed as dynamically interacting, resulting in cognitive performance and learning...

Increasingly, researchers have come to characterize cognition as a distributed process... [I]ntelligence can be seen as distributed in designed artifacts such as computer-user interfaces; in representations, such as diagrams; and through communication in social contexts... The idea of intelligence (i.e. knowledge and cognition) being distributed in a group, or in artifacts, customs, and situations, is interesting because it provides a framework for addressing a number of theoretical and empirical questions."

As discussed briefly, Patel and colleagues ([84,85] and references therein) analyze emergency room process and function from this perspective, finding what can be interpreted as 'rate distortion' error effects at shift change handover of patients between clinical teams, something emergency room personnel have characterized as a 'telephone game' [84]. This appears to be a prime example of a canonical failure mode involving rate-driven, distorted communication between institutional global workspaces.

Hutchins and collaborators have examined aircraft cockpits and naval vessels from similar perspectives, as have Woods and collaborators, and observed similar patterns.

### 3. Summary and a serious caution

We have outlined an approach to high order institutional cognition which appears consonant with much contemporary work on distributed cognition, although our treatment of resilience is far more general. What differentiates our analysis is the invocation of classes of necessary-conditions 'statistical' models based on the Rate Distortion Theorem, in the same sense that classes of empirical regression models are based on the Central Limit Theorem. All such models, while sometimes quite complicated, have a particular limited utility: the science does not lie within the models themselves, which are simply reexpressions of the asymptotic limit theorems of probability, but lies within comparisons between models fitted to the same system under different conditions, or between different systems facing similar conditions. The models we present here, like their regression near-kin, should be viewed as having been fitted to real systems, at least in theory, rather than as themselves underlying or defining those systems. These models do not, of themselves, cleave the Gordian Knot of scientific inference. That, as always, is a difficult matter of empirical and observational study. What we have done, however, is introduce a new quantitative toolkit which, when properly developed, may aid such study. Such development, however, remains to be done, so that particular sets of observations may be fitted to particular models. This kind of rigorous approach will require much time and very considerable resources. Nonetheless, as the next three sections show, qualitative inferences can be based on the modeling approach, and seem to provide important new insights to previous observations.

### 4. AIDS in Alameda County

A particularly striking example of institutional pathology in public health, representing a clear dysfunction of organizational collective consciousness, is the widespread failure of adequate response to AIDS in the United States. We examine three case histories. The first involves a geographic region near the principal West Coast epicenter. The second focuses on African-American religious groups in and near New York City, the principal East Coast epicenter. The third is a reconsideration of a broad, multi-city, case study of systematic failure in medical care provided to HIV positive women of color.

Alameda County lies on the east side of the San Francisco Bay from San Francisco, a virulent focus of the US AIDS pandemic, and is closely connected to it by the commuting field defined by the daily journey-to-work. Despite its proximity, Alameda County had very low rates of AIDS throughout the first decade of the epidemic, thus creating the opportunity for epidemic preparedness. The Epidemic Response in Alameda Study [100] was designed to learn how health care organizations just outside 'Ground Zero' for the American AIDS epidemic had responded to growing numbers of cases. The study used a three-stage epidemic response model, that started with recognition of an epidemic, garnering resources and realignment of social relationships to carry out epidemic-related tasks.

Health care agencies were enumerated using a variety of methods, including lists of agencies maintained by the County Department of Health, brochures of existing services and telephone books. A total of 51 agencies were identified. Directors of 31 agencies participated in the study by completing a semi-structured interview and filling out an agency information sheet. Interviews were carried out between March and December, 1989, well before introduction of antiretroviral drugs.

The recognition of the epidemic was slow and tortuous, slowed by stigma, lack of resources and lack of central leadership from federal, state or county agencies. Rather than the rapid mobilization of a system of organizations, what the study documented was the individual response of organizations that integrated new services as a result of a push-pull process. Among the pushes were: loss of key members of the organization to AIDS; growth of cases in the organization's service area; and the expansion of acknowledged 'affected' groups from gay men and intravenous drugs to include women, the elderly, and many others. Among the pulls were: new regulations imposed by government agencies, and new monies allotted for AIDS care. The directors were highly critical of the push-pull process, which left most organizations to their own devices. One director commented

"Just barely two years ago, I was coming over to [a community organization in San Francisco] and I was stealing buttons from [the director] and pamphlets and anything else I could steal. And I said, I have no shame because I have no money for AIDS and I wanted to bring that information but to my community here. So I did and, in fact, somebody interviewed me for the newspaper once and I said that I ran around and stole from everybody in San Francisco that I could steal from to bring information back to our community, until I got funding to do that. "

Garnering resources, the second stage of epidemic response, involved money as well as many other kinds of resources, like knowledge and connections. All of the organizations used existing resources at the beginning of their response to AIDS. As they began to understand the epidemic and carve out a path of action, all of the organizations looked for new resources to support new activities. At the level of the local hospitals, this involved massive expenditure for new forms of care. But even small organizations faced serious challenges relative to their existing and potential resources.

Central to the problems in garnering resources was the inadequate epidemic response by the society in general. Insufficient monies could not meet growing demands. Therefore, the monies were shifted from one group to another, using competition for funding to shift blame from government to providers. After all, if an agency failed to win a grant, it was the weakness of the application, not the overall lack of resources. In a funding-version of musical chairs, money moved among the interest groups, creating unnecessary strife and tension. One director noted,

"I, who could have worked on policy development, instead had to concentrate on funding. And spent a good year and half writing proposals and find funding sources and that kind of stuff. Thats part and parcel of doing business in the nonprofit world, but it took a year and a half to do that, whereas if we had been funded at the very start, I could have spent a lot more time trying to push forward our AIDS services as opposed to, first of all trying to educate funders about the Asian community, and then trying to find money for AIDS."

As the directors looked back on the eight years of epidemic response, it was clear that much social realignment had occurred, changing the social landscape of the county. New organizations were created, new coalitions were developed, and new personal relationships were established. One story captured the ways in which new contacts opened doors:

"There's a way in which it's helped people on my staff personally grow. To be more comfortable with gay people. I've one employee who's just the sweetest, most wonderful person, she's in her late 20s, has a wonderful home up in Castro Valley hills. We were talking one day about the story about the guy taking them out to the gay bar. She looked at me and she said, 'There's gay bars in Hayward? Really?' she says. She was shocked. She didn't know that. She'd never thought about it... It's gratifying to see people's openness and ability to be comfortable and welcoming, and to be touched by peoples stories and people's lives as much as they are by the homeless. "

On the basis of these data, the process of epidemic response in Alameda County was rated a failure. The study identified a large number of vulnerabilities that undermined epidemic response, including failure of multiple levels of government to provide leadership, widespread stigma, perhaps representing ideological rigidity associated with certain 'risk behaviors', and lack of an organized system of disease management that could easily incorporate new demands. Vulnerabilities are not typically considered in the planning of disaster response, yet it seems evident that this approach should be rethought. In fact, evidence from case studies of the crack epidemic, a fatal school shooting, the violence epidemic, and the response to Hurricanes Katrina and Rita suggests that vulnerabilities can easily derail response. Given the cost in human suffering the potential implications of organizational non-response to disaster should be given much more detailed examination.

These vulnerabilities, in particular resource limitations, and structurally-imposed Darwinian competition for available resources, have contributed to inattentional blindness/group denial, breakdown of intra-workspace communication (i.e. collaboration between different groups), and, ultimately, a systematic failure of engagement, possibly exacerbated by ideological constraints regarding 'unacceptable' risk behaviors. Although the weaknesses were clearly recognized, 'therapeutic' interventions against this pattern seem to have largely been ineffective for the very same reasons. This case history represents, paradoxically, an example of a pathological resilience – institutional inertia – in which deep-seated structural factors prevented effective recognition of, and response to, a significant threat.

### 5. AIDS and African American churches near New York City

Despite their excess risk for HIV/AIDS, African American communities have not developed a united response to this epidemic. Community-based organizations, especially those that provide addiction treatment services, have played an active role in developing HIV prevention and treatment services. Other organizations, however, have been more reticent to be involved; the churches are notable for their failure to support efforts at epidemic control, a finding which stands in marked contrast to the social activism that is commonly attributed to African-American clergy [101,102].

Like others in the mainstream of US society, African American clergy have watched from the sidelines of the epidemic. African Americans are closely linked to their clergy: 70 percent of those interviewed in a national survey reported they belong to a church and 71 percent report regular church attendance [102]. As in any community closely linked to its church, the role of pastors in setting social agendas reaches into nearly every aspect of life. Given the AIDS crisis in African American communities, the clergy's distance from epidemic response may be considered catastrophic.

Through their long history, African American churches have filled a dual mission in the community of ensuring physical survival while seeking spiritual salvation. African American churches were born out of the need to establish a place of worship that respected the humanity of African Americans. That struggle for personhood has never been far from the agenda of the churches, and the entry of the churches into the Civil Rights movement of the 1960's marked a turning point in that effort.

While many see the need for the churches to now lend their strength to the fight against AIDS, which has disproportionately affected African American communities, others – including many in the leadership of the churches – view AIDS as too closely linked to 'sinful' behavior to be an appropriate target for church activity. In contrast to the Civil Rights movement of the 1960's, which was consonant with the history and mission of the churches, the AIDS epidemic is sufficiently dissonant as to hamper institutional involvement.

A series of structured interviews with 51 African American clergy in and near New York City underlines this schism. The group had three particular characteristics. First, it was highly diverse. There is no single experience of church or religion, but rather many different ones, and this tradition of difference is fundamental. Second, the group prized their ability to interpret their holy books. Whereas in many areas of life, African Americans follow European Americans, in the realm of theology that is not the case. Third, the group was slow to respond to AIDS. While one or two individuals were active as early and 1984, the majority moved slowly and in response to the activities of others in the community, rather than being in the forefront themselves. AIDS has joined a long list of threats to community survival, including problems of poor education, loss of jobs, massive destruction of housing, and drug epidemics.

The clergy talked of AIDS in dire tones, but their sense of peril had not led to action. Rather, they were mired in a philosophical quandary over rescuing 'sinners' from the 'consequences' of their sins. Except for a handful who saw AIDS as a public health crisis, those interviewed linked HIV with 'sinful' behavior. They saw it as their duty to condemn sin; they might or might not display equal condemnation for the sinner.

The unwillingness to separate health and morality was at the heart of the hobbled effort to respond to AIDS. Clergy were ambivalent about what to do. Further, they were unwilling to take stands that might be controversial within their group. As one minister noted, pastors were reluctant to undertake AIDS activities because they feared others would say to the, "Why are you on the bandwagon for the gays?"

In spite of such constraints, however, at the time of the interviews, 1995, approximately two-thirds of the clergy interviewed had undertaken some form of AIDS activity. The incursion of AIDS into the day-to-day life of African Americans has thus forced many, though far from the majority, to confront the threat to life and health posed by the epidemic. In this context, the reality of death from AIDS has forced clergy to grapple with issues that challenge the key assumptions which undergird their ministries. Specifically, the majority of African American clergy view the behaviors that transmit AIDS as inextricably linked to moral concerns. Many cannot separate their role as moral arbiters from their other social and community roles. The concession to love the sinner but hate the sin is an unsatisfactory compromise that fails to create a climate of love and acceptance. Rather it feeds secrecy, shame and stigma, all of which inhibit AIDS prevention activities. The situation is accurately viewed as a doctrinal crisis.

Our findings underscore the extent to which, in the presence of uncertain danger and limited resources, as we have argued above in theory, ideology can become a rate-limiting factor for institutional response. Ideological struggles are rarely considered within the purview of public health. It is often quite difficult for those public health officials in the employ of a society divided along such lines to respond clearly and unequivocally to disease threat. Epidemic control is fundamentally a social action that requires the coordinated efforts of many people. When people are unable to agree on epidemic response, disease will spread. As we understand more about the political nature of epidemic propagation, we must alter and extend the practice of public health to take account of these realities.

### 6. Women's Equity in AIDS Resources

In 1999, the Health Resources and Services Administration (HRSA) commissioned five research teams to evaluate the extent to which the Ryan White CARE Act was meeting the needs of people living with AIDS. The Community Research Group, one of the teams, was asked to assess the effectiveness of the Ryan White program in meeting the needs of women of color living in 5 cities in the United States. The research team made site visits to 5 US cities, Los Angeles, Miami, New York, San Antonio and St. Louis. Interviews and observations and were conducted; proposals and other documents were reviewed [103,104].

At that time, a complex set of health and social services existed to provide a range of services to women of color living with HIV/AIDS. In each city, we found at least one place that had assembled a wide range of interconnected services, designed to meet the needs of poor women who had to manage not only their own health problems but also those of their children, other family members and friends. These "islands of success" as the research team dubbed them, understood that medical care needed to supplemented by such diverse services and supports as peer counseling, addiction treatment, housing aid, nutritional guidance, day care, and many others. This broad array of services was intended to and largely did meet the "web of needs" [105] of women living with the virus.

But, more striking than the existence of such islands of success was the relative scarcity of such holistic care, due, in large part, to the relative scarcity of some components of the package of services, and typically the shortage was in services that were needed by women but not men. A psychiatrist explained the kind of problems women faced:

"If you are a woman who is infected, and you have two kids. One is infected and one is not, but both are under the age of six. You have got to go to provider A for your primary care for your HIV. You might have to go to provider B for your outpatient substance abuse treatment that is on the west side of the county. Your infected child has to come here to the clinic for infected kids. But your affected child needs to go to a different provider, maybe the health department clinic, for his immunizations. One of those kids needs psychological services, and is being tested for developmental delay, a perfect example. The infected kid has a developmental delay secondary to the viral invasion of the central system. You as a woman have to orchestrate all of your service providers and all of the service providers for your kids, and you've got to haul them around almost every day. Plus youve got to go to the store, plus you've got to get your meds, plus you've got to go to the welfare department, plus, plus, plus It's mind boggling."

Not only did women have more needs, but also they had less power, education and money than men. One outreach worker commented,

"There is a huge gap, not only in survival rates, but in access to care and in understanding the treatments. Again, the men have had opportunities for education and jobs, they're more out in the world and they are more educated, so they can understand the information quicker than the women. Information that is not broken down in a way woman can relate to, and it is not women specific. The less women know about what is available to them, the less of a choice they have."

Decisions about the distribution of Ryan White Care dollars were influenced by community boards. Making sure that those boards acknowledged womens web of needs appeared to be an incompletely solved problem. As one interviewee put it:

"We need more representation of women on committees when different things are coming up and decisions are being made. Our health care is being based on how men have been taken care of in the past, and we are quite a bit different. We need to have a larger say-so on what is being decided."

Though women needed to be involved in the political process, they were hindered from doing so by cultural, financial and logistical problems. During a focus group at one clinic, a staff member pointed out, "Unfortunately, you can't bring in 10 infected women to stand up and make statements, they are just not ready to do that. And that is how a lot of gay men get services. Infected women don't have that type of voice."

A number of comments suggested that this inability to participate in the politics of AIDS care was culturally-based. One case manager observed:

"Women do not feel empowered. They do lots for their children and partners and put themselves last. There is culturally no power for women over sexuality in many communities. What should a woman do when sex and non-decisions are forced on her? Harm reduction must start young and women must be given power early. They need to be taught more about relationships and self-esteem."

Disempowerment was also related to the extenuating circumstances in which poor women of color often found themselves. A program coordinator at one center said:

"Minority women in treatment have a lot going on besides their HIV status. Most women as [our clinic] are the primary care takers of their families the primary income earners. They are undereducated and underemployed. They not only have to face disclosure issues, but also issues related to race and poverty. Daily, they stack all these issues on top of one another and sort out what they will address. At [our clinic], the women are allowed to incorporate all these things, whereas if they were just going to their doctor's office, they would not mention they don't have a phone. The staff at [our clinic] wants to know about things that are going on in their clients' daily lives and want to assist them in day-to-day activities. There are a lot of things that keep infected women from taking care of themselves. [Our clinic] tries to put daily living up front while helping women take time out for themselves."

Given an inadequately organized care system, women's web of needs was unlikely to be adequately addressed. This was not ameliorated by the system of community governance, which simply served to pit underserved communities against one another. In that system, women were at a great disadvantage, which worsened their situation.

Here resource limitation and competition, in concert with community disintegration, policy-driven limits causing service fragmentation, and very traditional, culturally-induced, inattentional blindness to the needs of poor women of color, combined synergistically to greatly undercut distributed institutional cognition affecting the provision of medical care and other essential services needed by this population.

### 7. Final remarks

One central outcome of our analysis has been an understanding that both social disintegration and resource limitation/competition can have much the same effect in degrading the ability of institutions to respond coherently to patterns of threat and opportunity, creating pathologies of inattentional blindness (IAB), failed internal communication and fragmentation (RD), and ideological or policy rigidity (PI). Such effects will be generated by imposed systems of draconian triage which limit institutional collective consciousness, trapping organizations, and their individual members, in pathological regimes of ecological resilience, various kinds of social eutrophication. Other, less subtle, historical paths, like disasters, produce similar results, which will be very resistant to change.

Communities, however, particularly constitute the unique riches of the poor, and those which are both socially disintegrated and poverty stricken will suffer synergistic burdens of institutional failure reflected in acute patterns of disease, violence, and substance abuse, which may be highly persistent.

Another central outcome has been the obvious role which internal organizational diversity – existence of multiple, different, global workspaces – can play in lessening the probability of organizational inattentional blindness. Many, very different foci, i.e. many different **R**_0_'s, can limit IAB, provided institutional rate distortion is modest – the different parts of the system talk to one another. The model is that 'many eyes can look in many directions'.

As a reviewer has noted, others have reached similar conclusions without the mathematical overhead. Woods and Hollnagel [63], for example, describe classic patterns of institutional error which appear to fit our model, including production pressure, fragmented problem solving, failure to revise assessments in the face of new evidence, and impaired communication and coordination. The reviewer particularly cited Woods' [106] comment on the Columbia space shuttle disaster: "What is striking is how there was a fragmented view of what was known about the strike and its potential implications over time, people, and groups. There was no place, artifact, or person who had a complete and coherent view of the analysis of the foam strike event".

Our work seems broadly consonant with mainstream research on distributed cognition, although our treatment of resilience is more informed by ecological than engineering paradigms, and our insistence on the central importance of culture in distributed cognition and institutional collective consciousness parallels continuing debates on ways culture affects individual psychology.

Responses to AIDS in the US provide many case histories in which imposed resource limitation and policy-driven social disintegration – structural violence – created institutional pathologies of denial, failed communication, and doctrinal inflexibility hindering epidemic response. These become rigidly locked-in, absent the massive infusion of resources and intensive community organizing which might enable social reintegration. Wallace and Wallace [89] and Fullilove [93], among others, show in some detail how relentless policies of serial forced displacement have gutted social organization within US minority communities, generating high rates of disease, substance abuse, and violence, while undercutting possible responses by institutions within the affected communities.

We end where we began. Humans in small, disciplined teams are the most fearsome predators on Earth. Partly, this is because the institutional collective consciousness of such groups, while not operating at the few hundred milliseconds of individual consciousness, is still very fast, while having both improved ability to act and decrease in the likelihood of being blindsided or suffering workspace failures. However institutional collective consciousness at larger scales seems far more complicated, and prone, as well, to particular errors and the vicissitudes of irreversible Lamarckian evolutionary process and pathological resilience.

Given the massive overt and structural violence of our times, these are not small matters, and our ability to manufacture vast social institutions, and the evident difficulties in correcting their pathologies, may, over the long term, ultimately be experienced as a seriously defective evolutionary adaptation.

## Mathematical appendix

### The Shannon-McMillan Theorem

According to the structure of the underlying language of which a message is a particular expression, some messages are more 'meaningful' than others, that is, are in accord with the grammar and syntax of the language. The Shannon-McMillan or Asymptotic Equipartition Theorem, describes how messages themselves are to be classified [33-35].

Suppose a long sequence of symbols is chosen, using the output of the random variable *X *above, so that an output sequence of length n, with the form

*x*_*n *_= (*α*_0_, *α*_1_, ..., *α*_*n*-1_)

has joint and conditional probabilities

*P*(*X*_0 _= *α*_0_, *X*_1 _= *α*_1_, ..., *X*_*n*-1 _= *α*_*n*-1_)

*P*(*X*_*n *_= *α*_*n*_|*X*_0 _= *α*_0_, ..., *X*_*n*-1 _= *α*_*n*-1_).     (21)

Using these probabilities we may calculate the conditional uncertainty

*H*(*X*_*n*_|*X*_0_, *X*_1_, ..., *X*_*n*-1_).

The uncertainty of the *information source*, *H*[**X**], is defined as

H[X]=lim⁡n→∞H(Xn|X0,X1,...,Xn−1).     (22)
 MathType@MTEF@5@5@+=feaafiart1ev1aaatCvAUfKttLearuWrP9MDH5MBPbIqV92AaeXatLxBI9gBaebbnrfifHhDYfgasaacH8akY=wiFfYdH8Gipec8Eeeu0xXdbba9frFj0=OqFfea0dXdd9vqai=hGuQ8kuc9pgc9s8qqaq=dirpe0xb9q8qiLsFr0=vr0=vr0dc8meaabaqaciaacaGaaeqabaqabeGadaaakeaacqWGibascqGGBbWwieqacqWFybawcqGGDbqxcqGH9aqpdaWfqaqaaiGbcYgaSjabcMgaPjabc2gaTbWcbaGaemOBa4MaeyOKH4QaeyOhIukabeaakiabdIeaijabcIcaOiabdIfaynaaBaaaleaacqWGUbGBaeqaaOGaeiiFaWNaemiwaG1aaSbaaSqaaiabicdaWaqabaGccqGGSaalcqWGybawdaWgaaWcbaGaeGymaedabeaakiabcYcaSiabc6caUiabc6caUiabc6caUiabcYcaSiabdIfaynaaBaaaleaacqWGUbGBcqGHsislcqaIXaqmaeqaaOGaeiykaKIaeiOla4IaaCzcaiaaxMaadaqadaqaaiabikdaYiabikdaYaGaayjkaiaawMcaaaaa@571B@

In general

*H*(*X*_*n*_|*X*_0_, *X*_1_, ..., *X*_*n*-1_) ≤ *H*(*X*_*n*_).

Only if the random variables *X*_*j *_are all stochastically independent does equality hold. If there is a maximum *n *such that, for all *m *> 0

*H*(*X*_*n*+*m*_|*X*_0_, ..., *X*_*n*+*m*-1 _= *H*(*X*_*n*_|*X*_0_, ..., *X*_*n*-1_),

then the source is said to be of *order *n. It is easy to show that

H[X]=lim⁡n→∞H(X0,...Xn)n+1.
 MathType@MTEF@5@5@+=feaafiart1ev1aaatCvAUfKttLearuWrP9MDH5MBPbIqV92AaeXatLxBI9gBaebbnrfifHhDYfgasaacH8akY=wiFfYdH8Gipec8Eeeu0xXdbba9frFj0=OqFfea0dXdd9vqai=hGuQ8kuc9pgc9s8qqaq=dirpe0xb9q8qiLsFr0=vr0=vr0dc8meaabaqaciaacaGaaeqabaqabeGadaaakeaacqWGibascqGGBbWwieqacqWFybawcqGGDbqxcqGH9aqpdaWfqaqaaiGbcYgaSjabcMgaPjabc2gaTbWcbaGaemOBa4MaeyOKH4QaeyOhIukabeaakmaalaaabaGaemisaGKaeiikaGIaemiwaG1aaSbaaSqaaiabicdaWaqabaGccqGGSaalcqGGUaGlcqGGUaGlcqGGUaGlcqWGybawdaWgaaWcbaGaemOBa4gabeaakiabcMcaPaqaaiabd6gaUjabgUcaRiabigdaXaaacqGGUaGlaaa@4B61@

In general the outputs of the *X*_*j*_, *j *= 0, 1,..., *n *are *dependent*. That is, the output of the communication process at step *n *depends on previous steps. Such serial correlation, in fact, is the very structure which enables most of what is done in this paper.

Here, however, the processes are all assumed stationary in time, that is, the serial correlations do not change in time, and the system is *stationary*.

A very broad class of such self-correlated, stationary, information sources, the so-called *ergodic *sources for which the long-run relative frequency of a sequence converges stochastically to the probability assigned to it, have a particularly interesting property:

It is possible, in the limit of large *n*, to divide all sequences of outputs of an ergodic information source into two distinct sets, *S*_1 _and *S*_2_, having, respectively, very high and very low probabilities of occurrence, with the source uncertainty providing the splitting criterion. In particular the Shannon-McMillan Theorem states that, for a (long) sequence having *n *(serially correlated) elements, the number of 'meaningful' sequences, *N*(*n*) – those belonging to set *S*_1 _– will satisfy the relation

log⁡[N(n)]n≈H[X].     (23)
 MathType@MTEF@5@5@+=feaafiart1ev1aaatCvAUfKttLearuWrP9MDH5MBPbIqV92AaeXatLxBI9gBaebbnrfifHhDYfgasaacH8akY=wiFfYdH8Gipec8Eeeu0xXdbba9frFj0=OqFfea0dXdd9vqai=hGuQ8kuc9pgc9s8qqaq=dirpe0xb9q8qiLsFr0=vr0=vr0dc8meaabaqaciaacaGaaeqabaqabeGadaaakeaadaWcaaqaaiGbcYgaSjabc+gaVjabcEgaNjabcUfaBjabd6eaojabcIcaOiabd6gaUjabcMcaPiabc2faDbqaaiabd6gaUbaacqGHijYUcqWGibascqGGBbWwieqacqWFybawcqGGDbqxcqGGUaGlcaWLjaGaaCzcamaabmaabaGaeGOmaiJaeG4mamdacaGLOaGaayzkaaaaaa@451B@

More formally,

lim⁡n→∞log⁡[N(n)]n=H[X]=lim⁡n→∞H(Xn|X0,...,Xn−1)=lim⁡n→∞H(X0,...,Xn)n+1.     (24)
 MathType@MTEF@5@5@+=feaafiart1ev1aaatCvAUfKttLearuWrP9MDH5MBPbIqV92AaeXatLxBI9gBaebbnrfifHhDYfgasaacH8akY=wiFfYdH8Gipec8Eeeu0xXdbba9frFj0=OqFfea0dXdd9vqai=hGuQ8kuc9pgc9s8qqaq=dirpe0xb9q8qiLsFr0=vr0=vr0dc8meaabaqaciaacaGaaeqabaqabeGadaaakeaafaqabeWabaaabaWaaCbeaeaacyGGSbaBcqGGPbqAcqGGTbqBaSqaaiabd6gaUjabgkziUkabg6HiLcqabaGcdaWcaaqaaiGbcYgaSjabc+gaVjabcEgaNjabcUfaBjabd6eaojabcIcaOiabd6gaUjabcMcaPiabc2faDbqaaiabd6gaUbaacqGH9aqpcqWGibascqGGBbWwieqacqWFybawcqGGDbqxaeaacqGH9aqpdaWfqaqaaiGbcYgaSjabcMgaPjabc2gaTbWcbaGaemOBa4MaeyOKH4QaeyOhIukabeaakiabdIeaijabcIcaOiabdIfaynaaBaaaleaacqWGUbGBaeqaaOGaeiiFaWNaemiwaG1aaSbaaSqaaiabicdaWaqabaGccqGGSaalcqGGUaGlcqGGUaGlcqGGUaGlcqGGSaalcqWGybawdaWgaaWcbaGaemOBa4MaeyOeI0IaeGymaedabeaakiabcMcaPaqaaiabg2da9maaxababaGagiiBaWMaeiyAaKMaeiyBa0galeaacqWGUbGBcqGHsgIRcqGHEisPaeqaaOWaaSaaaeaacqWGibascqGGOaakcqWGybawdaWgaaWcbaGaeGimaadabeaakiabcYcaSiabc6caUiabc6caUiabc6caUiabcYcaSiabdIfaynaaBaaaleaacqWGUbGBaeqaaOGaeiykaKcabaGaemOBa4Maey4kaSIaeGymaedaaiabc6caUaaacaWLjaGaaCzcamaabmaabaGaeGOmaiJaeGinaqdacaGLOaGaayzkaaaaaa@8441@

Using the internal structures of the information source permits *limiting attention only to high probability 'meaningful' sequences of symbols*.

### The Rate Distortion Theorem

The Shannon-McMillan Theorem can be expressed as the 'zero error limit' of the Rate Distortion Theorem [35,107], which defines a splitting criterion that identifies high probability pairs of sequences. We follow closely the treatment of [35].

The origin of the problem is the question of representing one information source by a simpler one in such a way that the least information is lost. For example we might have a continuous variate between 0 and 100, and wish to represent it in terms of a small set of integers in a way that minimizes the inevitable distortion that process creates. Typically, for example, an analog audio signal will be replaced by a 'digital' one. The problem is to do this in a way which least distorts the *reconstructed *audio waveform.

Suppose the original stationary, ergodic information source *Y *with output from a particular alphabet generates sequences of the form

*y*^*n *^= *y*_1_, ..., *y*_*n*_.

These are 'digitized,' in some sense, producing a chain of 'digitized values'

*b*^*n *^= *b*_1_, ..., *b*_*n*_,

where the *b*-alphabet is much more restricted than the *y*-alphabet.

*b*^*n *^is, in turn, *deterministically retranslated *into a reproduction of the original signal *y*^*n*^. That is, each *b*^*m *^is mapped on to a unique n-length y-sequence in the alphabet of the information source *Y*:

bm→y^n=y^1,...,y^n.
 MathType@MTEF@5@5@+=feaafiart1ev1aaatCvAUfKttLearuWrP9MDH5MBPbIqV92AaeXatLxBI9gBaebbnrfifHhDYfgasaacH8akY=wiFfYdH8Gipec8Eeeu0xXdbba9frFj0=OqFfea0dXdd9vqai=hGuQ8kuc9pgc9s8qqaq=dirpe0xb9q8qiLsFr0=vr0=vr0dc8meaabaqaciaacaGaaeqabaqabeGadaaakeaacqWGIbGydaahaaWcbeqaaiabd2gaTbaakiabgkziUkqbdMha5zaajaWaaWbaaSqabeaacqWGUbGBaaGccqGH9aqpcuWG5bqEgaqcamaaBaaaleaacqaIXaqmaeqaaOGaeiilaWIaeiOla4IaeiOla4IaeiOla4IaeiilaWIafmyEaKNbaKaadaWgaaWcbaGaemOBa4gabeaakiabc6caUaaa@40D4@

Note, however, that many *y*^*n *^sequences may be mapped onto the *same *retranslation sequence y^
 MathType@MTEF@5@5@+=feaafiart1ev1aaatCvAUfKttLearuWrP9MDH5MBPbIqV92AaeXatLxBI9gBaebbnrfifHhDYfgasaacH8akY=wiFfYdH8Gipec8Eeeu0xXdbba9frFj0=OqFfea0dXdd9vqai=hGuQ8kuc9pgc9s8qqaq=dirpe0xb9q8qiLsFr0=vr0=vr0dc8meaabaqaciaacaGaaeqabaqabeGadaaakeaacuWG5bqEgaqcaaaa@2E37@^*n*^, so that information will, in general, be lost.

The central problem is to explicitly minimize that loss.

The retranslation process defines a new stationary, ergodic information source, Y^
 MathType@MTEF@5@5@+=feaafiart1ev1aaatCvAUfKttLearuWrP9MDH5MBPbIqV92AaeXatLxBI9gBaebbnrfifHhDYfgasaacH8akY=wiFfYdH8Gipec8Eeeu0xXdbba9frFj0=OqFfea0dXdd9vqai=hGuQ8kuc9pgc9s8qqaq=dirpe0xb9q8qiLsFr0=vr0=vr0dc8meaabaqaciaacaGaaeqabaqabeGadaaakeaacuWGzbqwgaqcaaaa@2DF7@.

The next step is to define a *distortion measure*, *d*(*y*, y^
 MathType@MTEF@5@5@+=feaafiart1ev1aaatCvAUfKttLearuWrP9MDH5MBPbIqV92AaeXatLxBI9gBaebbnrfifHhDYfgasaacH8akY=wiFfYdH8Gipec8Eeeu0xXdbba9frFj0=OqFfea0dXdd9vqai=hGuQ8kuc9pgc9s8qqaq=dirpe0xb9q8qiLsFr0=vr0=vr0dc8meaabaqaciaacaGaaeqabaqabeGadaaakeaacuWG5bqEgaqcaaaa@2E37@), which compares the original to the retranslated path. For example the *Hamming distortion *is

*d*(*y*, y^
 MathType@MTEF@5@5@+=feaafiart1ev1aaatCvAUfKttLearuWrP9MDH5MBPbIqV92AaeXatLxBI9gBaebbnrfifHhDYfgasaacH8akY=wiFfYdH8Gipec8Eeeu0xXdbba9frFj0=OqFfea0dXdd9vqai=hGuQ8kuc9pgc9s8qqaq=dirpe0xb9q8qiLsFr0=vr0=vr0dc8meaabaqaciaacaGaaeqabaqabeGadaaakeaacuWG5bqEgaqcaaaa@2E37@) = 1, *y *≠ y^
 MathType@MTEF@5@5@+=feaafiart1ev1aaatCvAUfKttLearuWrP9MDH5MBPbIqV92AaeXatLxBI9gBaebbnrfifHhDYfgasaacH8akY=wiFfYdH8Gipec8Eeeu0xXdbba9frFj0=OqFfea0dXdd9vqai=hGuQ8kuc9pgc9s8qqaq=dirpe0xb9q8qiLsFr0=vr0=vr0dc8meaabaqaciaacaGaaeqabaqabeGadaaakeaacuWG5bqEgaqcaaaa@2E37@

*d*(*y*, y^
 MathType@MTEF@5@5@+=feaafiart1ev1aaatCvAUfKttLearuWrP9MDH5MBPbIqV92AaeXatLxBI9gBaebbnrfifHhDYfgasaacH8akY=wiFfYdH8Gipec8Eeeu0xXdbba9frFj0=OqFfea0dXdd9vqai=hGuQ8kuc9pgc9s8qqaq=dirpe0xb9q8qiLsFr0=vr0=vr0dc8meaabaqaciaacaGaaeqabaqabeGadaaakeaacuWG5bqEgaqcaaaa@2E37@) = 0, *y *= y^
 MathType@MTEF@5@5@+=feaafiart1ev1aaatCvAUfKttLearuWrP9MDH5MBPbIqV92AaeXatLxBI9gBaebbnrfifHhDYfgasaacH8akY=wiFfYdH8Gipec8Eeeu0xXdbba9frFj0=OqFfea0dXdd9vqai=hGuQ8kuc9pgc9s8qqaq=dirpe0xb9q8qiLsFr0=vr0=vr0dc8meaabaqaciaacaGaaeqabaqabeGadaaakeaacuWG5bqEgaqcaaaa@2E37@.     (25)

For continuous variates the *Squared error distortion *is

*d*(*y*, y^
 MathType@MTEF@5@5@+=feaafiart1ev1aaatCvAUfKttLearuWrP9MDH5MBPbIqV92AaeXatLxBI9gBaebbnrfifHhDYfgasaacH8akY=wiFfYdH8Gipec8Eeeu0xXdbba9frFj0=OqFfea0dXdd9vqai=hGuQ8kuc9pgc9s8qqaq=dirpe0xb9q8qiLsFr0=vr0=vr0dc8meaabaqaciaacaGaaeqabaqabeGadaaakeaacuWG5bqEgaqcaaaa@2E37@) = (*y *- y^
 MathType@MTEF@5@5@+=feaafiart1ev1aaatCvAUfKttLearuWrP9MDH5MBPbIqV92AaeXatLxBI9gBaebbnrfifHhDYfgasaacH8akY=wiFfYdH8Gipec8Eeeu0xXdbba9frFj0=OqFfea0dXdd9vqai=hGuQ8kuc9pgc9s8qqaq=dirpe0xb9q8qiLsFr0=vr0=vr0dc8meaabaqaciaacaGaaeqabaqabeGadaaakeaacuWG5bqEgaqcaaaa@2E37@)^2^.     (26)

There are many possibilities.

The distortion between paths *y*^*n *^and y^
 MathType@MTEF@5@5@+=feaafiart1ev1aaatCvAUfKttLearuWrP9MDH5MBPbIqV92AaeXatLxBI9gBaebbnrfifHhDYfgasaacH8akY=wiFfYdH8Gipec8Eeeu0xXdbba9frFj0=OqFfea0dXdd9vqai=hGuQ8kuc9pgc9s8qqaq=dirpe0xb9q8qiLsFr0=vr0=vr0dc8meaabaqaciaacaGaaeqabaqabeGadaaakeaacuWG5bqEgaqcaaaa@2E37@^*n*^is defined as

f(yn,y^n)=1n∑j=1nd(yj,y^j).     (27)
 MathType@MTEF@5@5@+=feaafiart1ev1aaatCvAUfKttLearuWrP9MDH5MBPbIqV92AaeXatLxBI9gBaebbnrfifHhDYfgasaacH8akY=wiFfYdH8Gipec8Eeeu0xXdbba9frFj0=OqFfea0dXdd9vqai=hGuQ8kuc9pgc9s8qqaq=dirpe0xb9q8qiLsFr0=vr0=vr0dc8meaabaqaciaacaGaaeqabaqabeGadaaakeaacqWGMbGzcqGGOaakcqWG5bqEdaahaaWcbeqaaiabd6gaUbaakiabcYcaSiqbdMha5zaajaWaaWbaaSqabeaacqWGUbGBaaGccqGGPaqkcqGH9aqpdaWcaaqaaiabigdaXaqaaiabd6gaUbaadaaeWbqaaiabdsgaKjabcIcaOiabdMha5naaBaaaleaacqWGQbGAaeqaaOGaeiilaWIafmyEaKNbaKaadaWgaaWcbaGaemOAaOgabeaakiabcMcaPaWcbaGaemOAaOMaeyypa0JaeGymaedabaGaemOBa4ganiabggHiLdGccqGGUaGlcaWLjaGaaCzcamaabmaabaGaeGOmaiJaeG4naCdacaGLOaGaayzkaaaaaa@50EE@

Suppose that with each path *y*^*n *^and *b*^*n*^-path retranslation into the *y*-language and denoted *y*^*n*^, there are associated individual, joint, and conditional probability distributions

*p*(*y*^*n*^), *p*(y^
 MathType@MTEF@5@5@+=feaafiart1ev1aaatCvAUfKttLearuWrP9MDH5MBPbIqV92AaeXatLxBI9gBaebbnrfifHhDYfgasaacH8akY=wiFfYdH8Gipec8Eeeu0xXdbba9frFj0=OqFfea0dXdd9vqai=hGuQ8kuc9pgc9s8qqaq=dirpe0xb9q8qiLsFr0=vr0=vr0dc8meaabaqaciaacaGaaeqabaqabeGadaaakeaacuWG5bqEgaqcaaaa@2E37@^*n*^), *p*(*y*^*n*^|y^
 MathType@MTEF@5@5@+=feaafiart1ev1aaatCvAUfKttLearuWrP9MDH5MBPbIqV92AaeXatLxBI9gBaebbnrfifHhDYfgasaacH8akY=wiFfYdH8Gipec8Eeeu0xXdbba9frFj0=OqFfea0dXdd9vqai=hGuQ8kuc9pgc9s8qqaq=dirpe0xb9q8qiLsFr0=vr0=vr0dc8meaabaqaciaacaGaaeqabaqabeGadaaakeaacuWG5bqEgaqcaaaa@2E37@^*n*^).

The *average distortion *is defined as

D=∑ynp(yn)d(yn,y^n).     (28)
 MathType@MTEF@5@5@+=feaafiart1ev1aaatCvAUfKttLearuWrP9MDH5MBPbIqV92AaeXatLxBI9gBaebbnrfifHhDYfgasaacH8akY=wiFfYdH8Gipec8Eeeu0xXdbba9frFj0=OqFfea0dXdd9vqai=hGuQ8kuc9pgc9s8qqaq=dirpe0xb9q8qiLsFr0=vr0=vr0dc8meaabaqaciaacaGaaeqabaqabeGadaaakeaacqWGebarcqGH9aqpdaaeqbqaaiabdchaWjabcIcaOiabdMha5naaCaaaleqabaGaemOBa4gaaOGaeiykaKIaemizaqMaeiikaGIaemyEaK3aaWbaaSqabeaacqWGUbGBaaGccqGGSaalcuWG5bqEgaqcamaaCaaaleqabaGaemOBa4gaaOGaeiykaKcaleaacqWG5bqEdaahaaadbeqaaiabd6gaUbaaaSqab0GaeyyeIuoakiabc6caUiaaxMaacaWLjaWaaeWaaeaacqaIYaGmcqaI4aaoaiaawIcacaGLPaaaaaa@49FD@

It is possible, using the distributions given above, to define the information transmitted from the incoming *Y *to the outgoing Y^
 MathType@MTEF@5@5@+=feaafiart1ev1aaatCvAUfKttLearuWrP9MDH5MBPbIqV92AaeXatLxBI9gBaebbnrfifHhDYfgasaacH8akY=wiFfYdH8Gipec8Eeeu0xXdbba9frFj0=OqFfea0dXdd9vqai=hGuQ8kuc9pgc9s8qqaq=dirpe0xb9q8qiLsFr0=vr0=vr0dc8meaabaqaciaacaGaaeqabaqabeGadaaakeaacuWGzbqwgaqcaaaa@2DF7@ process in the usual manner, using the Shannon source uncertainty of the strings:

*I*(*Y*, Y^
 MathType@MTEF@5@5@+=feaafiart1ev1aaatCvAUfKttLearuWrP9MDH5MBPbIqV92AaeXatLxBI9gBaebbnrfifHhDYfgasaacH8akY=wiFfYdH8Gipec8Eeeu0xXdbba9frFj0=OqFfea0dXdd9vqai=hGuQ8kuc9pgc9s8qqaq=dirpe0xb9q8qiLsFr0=vr0=vr0dc8meaabaqaciaacaGaaeqabaqabeGadaaakeaacuWGzbqwgaqcaaaa@2DF7@) ≡ *H*(*Y*) - *H*(*Y*|Y^
 MathType@MTEF@5@5@+=feaafiart1ev1aaatCvAUfKttLearuWrP9MDH5MBPbIqV92AaeXatLxBI9gBaebbnrfifHhDYfgasaacH8akY=wiFfYdH8Gipec8Eeeu0xXdbba9frFj0=OqFfea0dXdd9vqai=hGuQ8kuc9pgc9s8qqaq=dirpe0xb9q8qiLsFr0=vr0=vr0dc8meaabaqaciaacaGaaeqabaqabeGadaaakeaacuWGzbqwgaqcaaaa@2DF7@) = *H*(*Y*) + *H*(Y^
 MathType@MTEF@5@5@+=feaafiart1ev1aaatCvAUfKttLearuWrP9MDH5MBPbIqV92AaeXatLxBI9gBaebbnrfifHhDYfgasaacH8akY=wiFfYdH8Gipec8Eeeu0xXdbba9frFj0=OqFfea0dXdd9vqai=hGuQ8kuc9pgc9s8qqaq=dirpe0xb9q8qiLsFr0=vr0=vr0dc8meaabaqaciaacaGaaeqabaqabeGadaaakeaacuWGzbqwgaqcaaaa@2DF7@) - *H*(*Y*, Y^
 MathType@MTEF@5@5@+=feaafiart1ev1aaatCvAUfKttLearuWrP9MDH5MBPbIqV92AaeXatLxBI9gBaebbnrfifHhDYfgasaacH8akY=wiFfYdH8Gipec8Eeeu0xXdbba9frFj0=OqFfea0dXdd9vqai=hGuQ8kuc9pgc9s8qqaq=dirpe0xb9q8qiLsFr0=vr0=vr0dc8meaabaqaciaacaGaaeqabaqabeGadaaakeaacuWGzbqwgaqcaaaa@2DF7@).

If there is no uncertainty in *Y *given the retranslation Y^
 MathType@MTEF@5@5@+=feaafiart1ev1aaatCvAUfKttLearuWrP9MDH5MBPbIqV92AaeXatLxBI9gBaebbnrfifHhDYfgasaacH8akY=wiFfYdH8Gipec8Eeeu0xXdbba9frFj0=OqFfea0dXdd9vqai=hGuQ8kuc9pgc9s8qqaq=dirpe0xb9q8qiLsFr0=vr0=vr0dc8meaabaqaciaacaGaaeqabaqabeGadaaakeaacuWGzbqwgaqcaaaa@2DF7@, then no information is lost.

In general, this will not be true.

The *information rate distortion function R*(*D*) for a source *Y *with a distortion measure *d*(*y*, y^
 MathType@MTEF@5@5@+=feaafiart1ev1aaatCvAUfKttLearuWrP9MDH5MBPbIqV92AaeXatLxBI9gBaebbnrfifHhDYfgasaacH8akY=wiFfYdH8Gipec8Eeeu0xXdbba9frFj0=OqFfea0dXdd9vqai=hGuQ8kuc9pgc9s8qqaq=dirpe0xb9q8qiLsFr0=vr0=vr0dc8meaabaqaciaacaGaaeqabaqabeGadaaakeaacuWG5bqEgaqcaaaa@2E37@) is defined as

R(D)=min⁡p(y,y^);∑(y,y^)p(y)p(y|y^)d(y,y^)≤DI(Y,Y^).     (29)
 MathType@MTEF@5@5@+=feaafiart1ev1aaatCvAUfKttLearuWrP9MDH5MBPbIqV92AaeXatLxBI9gBaebbnrfifHhDYfgasaacH8akY=wiFfYdH8Gipec8Eeeu0xXdbba9frFj0=OqFfea0dXdd9vqai=hGuQ8kuc9pgc9s8qqaq=dirpe0xb9q8qiLsFr0=vr0=vr0dc8meaabaqaciaacaGaaeqabaqabeGadaaakeaacqWGsbGucqGGOaakcqWGebarcqGGPaqkcqGH9aqpdaWfqaqaaiGbc2gaTjabcMgaPjabc6gaUbWcbaGaemiCaaNaeiikaGIaemyEaKNaeiilaWIafmyEaKNbaKaacqGGPaqkcqGG7aWodaaeqaqaaiabdchaWjabcIcaOiabdMha5jabcMcaPiabdchaWjabcIcaOiabdMha5jabcYha8jqbdMha5zaajaGaeiykaKIaemizaqMaeiikaGIaemyEaKNaeiilaWIafmyEaKNbaKaacqGGPaqkcqGHKjYOcqWGebaraWqaaiabcIcaOiabdMha5jabcYcaSiqbdMha5zaajaGaeiykaKcabeGdcqGHris5aaWcbeaakiabdMeajjabcIcaOiabdMfazjabcYcaSiqbdMfazzaajaGaeiykaKIaeiOla4IaaCzcaiaaxMaadaqadaqaaiabikdaYiabiMda5aGaayjkaiaawMcaaaaa@674A@

The minimization is over all conditional distributions *p*(*y*|y^
 MathType@MTEF@5@5@+=feaafiart1ev1aaatCvAUfKttLearuWrP9MDH5MBPbIqV92AaeXatLxBI9gBaebbnrfifHhDYfgasaacH8akY=wiFfYdH8Gipec8Eeeu0xXdbba9frFj0=OqFfea0dXdd9vqai=hGuQ8kuc9pgc9s8qqaq=dirpe0xb9q8qiLsFr0=vr0=vr0dc8meaabaqaciaacaGaaeqabaqabeGadaaakeaacuWG5bqEgaqcaaaa@2E37@) for which the joint distribution *p*(*y*, y^
 MathType@MTEF@5@5@+=feaafiart1ev1aaatCvAUfKttLearuWrP9MDH5MBPbIqV92AaeXatLxBI9gBaebbnrfifHhDYfgasaacH8akY=wiFfYdH8Gipec8Eeeu0xXdbba9frFj0=OqFfea0dXdd9vqai=hGuQ8kuc9pgc9s8qqaq=dirpe0xb9q8qiLsFr0=vr0=vr0dc8meaabaqaciaacaGaaeqabaqabeGadaaakeaacuWG5bqEgaqcaaaa@2E37@) = *p*(*y*)*p*(*y*|y^
 MathType@MTEF@5@5@+=feaafiart1ev1aaatCvAUfKttLearuWrP9MDH5MBPbIqV92AaeXatLxBI9gBaebbnrfifHhDYfgasaacH8akY=wiFfYdH8Gipec8Eeeu0xXdbba9frFj0=OqFfea0dXdd9vqai=hGuQ8kuc9pgc9s8qqaq=dirpe0xb9q8qiLsFr0=vr0=vr0dc8meaabaqaciaacaGaaeqabaqabeGadaaakeaacuWG5bqEgaqcaaaa@2E37@) satisfies the average distortion constraint (i.e. average distortion ≤ *D*).

The *Rate Distortion Theorem *states that *R*(*D*) *is the maximum achievable rate of information transmission which does not exceed the distortion D*. References [35,107] provide details.

More to the point, however, is the following: Pairs of sequences (*y*^*n*^, y^
 MathType@MTEF@5@5@+=feaafiart1ev1aaatCvAUfKttLearuWrP9MDH5MBPbIqV92AaeXatLxBI9gBaebbnrfifHhDYfgasaacH8akY=wiFfYdH8Gipec8Eeeu0xXdbba9frFj0=OqFfea0dXdd9vqai=hGuQ8kuc9pgc9s8qqaq=dirpe0xb9q8qiLsFr0=vr0=vr0dc8meaabaqaciaacaGaaeqabaqabeGadaaakeaacuWG5bqEgaqcaaaa@2E37@^*n*^) can be defined as *distortion typical*; that is, for a given average distortion *D*, defined in terms of a particular measure, pairs of sequences can be divided into two sets, a high probability one containing a relatively small number of (matched) pairs with *d*(*y*^*n*^, y^
 MathType@MTEF@5@5@+=feaafiart1ev1aaatCvAUfKttLearuWrP9MDH5MBPbIqV92AaeXatLxBI9gBaebbnrfifHhDYfgasaacH8akY=wiFfYdH8Gipec8Eeeu0xXdbba9frFj0=OqFfea0dXdd9vqai=hGuQ8kuc9pgc9s8qqaq=dirpe0xb9q8qiLsFr0=vr0=vr0dc8meaabaqaciaacaGaaeqabaqabeGadaaakeaacuWG5bqEgaqcaaaa@2E37@^*n*^) ≤ *D*, and a low probability one containing most pairs. As *n *→ ∞, the smaller set approaches unit probability, and, for those pairs,

*p*(*y*^*n*^) ≥ *p*(y^
 MathType@MTEF@5@5@+=feaafiart1ev1aaatCvAUfKttLearuWrP9MDH5MBPbIqV92AaeXatLxBI9gBaebbnrfifHhDYfgasaacH8akY=wiFfYdH8Gipec8Eeeu0xXdbba9frFj0=OqFfea0dXdd9vqai=hGuQ8kuc9pgc9s8qqaq=dirpe0xb9q8qiLsFr0=vr0=vr0dc8meaabaqaciaacaGaaeqabaqabeGadaaakeaacuWG5bqEgaqcaaaa@2E37@^*n*^|*y*^*n*^) exp[-*nI*(*Y*, Y^
 MathType@MTEF@5@5@+=feaafiart1ev1aaatCvAUfKttLearuWrP9MDH5MBPbIqV92AaeXatLxBI9gBaebbnrfifHhDYfgasaacH8akY=wiFfYdH8Gipec8Eeeu0xXdbba9frFj0=OqFfea0dXdd9vqai=hGuQ8kuc9pgc9s8qqaq=dirpe0xb9q8qiLsFr0=vr0=vr0dc8meaabaqaciaacaGaaeqabaqabeGadaaakeaacuWGzbqwgaqcaaaa@2DF7@)]

Thus, roughly speaking, *I*(*Y*, Y^
 MathType@MTEF@5@5@+=feaafiart1ev1aaatCvAUfKttLearuWrP9MDH5MBPbIqV92AaeXatLxBI9gBaebbnrfifHhDYfgasaacH8akY=wiFfYdH8Gipec8Eeeu0xXdbba9frFj0=OqFfea0dXdd9vqai=hGuQ8kuc9pgc9s8qqaq=dirpe0xb9q8qiLsFr0=vr0=vr0dc8meaabaqaciaacaGaaeqabaqabeGadaaakeaacuWGzbqwgaqcaaaa@2DF7@) embodies the splitting criterion between high and low probability pairs of paths.

For the theory of interacting information sources, then, *I*(*Y*, Y^
 MathType@MTEF@5@5@+=feaafiart1ev1aaatCvAUfKttLearuWrP9MDH5MBPbIqV92AaeXatLxBI9gBaebbnrfifHhDYfgasaacH8akY=wiFfYdH8Gipec8Eeeu0xXdbba9frFj0=OqFfea0dXdd9vqai=hGuQ8kuc9pgc9s8qqaq=dirpe0xb9q8qiLsFr0=vr0=vr0dc8meaabaqaciaacaGaaeqabaqabeGadaaakeaacuWGzbqwgaqcaaaa@2DF7@) can play the role of *H *in the dynamic treatment that follows.

The rate distortion function can actually be calculated in many cases by using a Lagrange multiplier method – see Section 13.7 of [35].
